# Vesicular trafficking and cell-cell communication in neurodevelopment and neurodegeneration

**DOI:** 10.3389/fcell.2025.1600034

**Published:** 2025-06-09

**Authors:** Salma Amin, Elena Taverna

**Affiliations:** Human Technopole, Milan, Italy

**Keywords:** golgi apparatus, neurons, lysosomes, retrograde and anterograde transport, neural stem cells, brain development

## Abstract

Regulation of vesicle biology and trafficking plays a critical role in cell viability. Vesicular trafficking is a process that entails vesicle biogenesis, transport, and sorting of materials such as proteins, enzymes, hormones, and neurotransmitters to different cellular compartments. This phenomenon is especially important in cells of the central nervous system, including neural progenitors, neurons, and glial cell populations, because of their highly polarized architecture. In line with that, disruption in vesicular trafficking during cortical development affects progenitor proliferation and differentiation and leads to brain malformations. On the other hand, neuronal cells require long-range vesicular trafficking to reach distant locations, such as the distal part of the axons, and synaptic vesicles are essential for cell-cell communication. Neurons have high energy demands. Therefore, any malfunction in vesicular trafficking is a trigger to spiraling into neurodegeneration. Here, we give a comprehensive review of the role of intracellular and extracellular vesicles in cortical development and neurodegeneration, and we discuss how trafficking between organelles in specific cell types contributes to brain pathologies. Finally, we highlight the emerging evidence linking disruption in vesicular trafficking to neurological disorders such as Alzheimer's disease, Parkinson's disease, and autism.

## 1 Introduction

**FIGURE 1:**
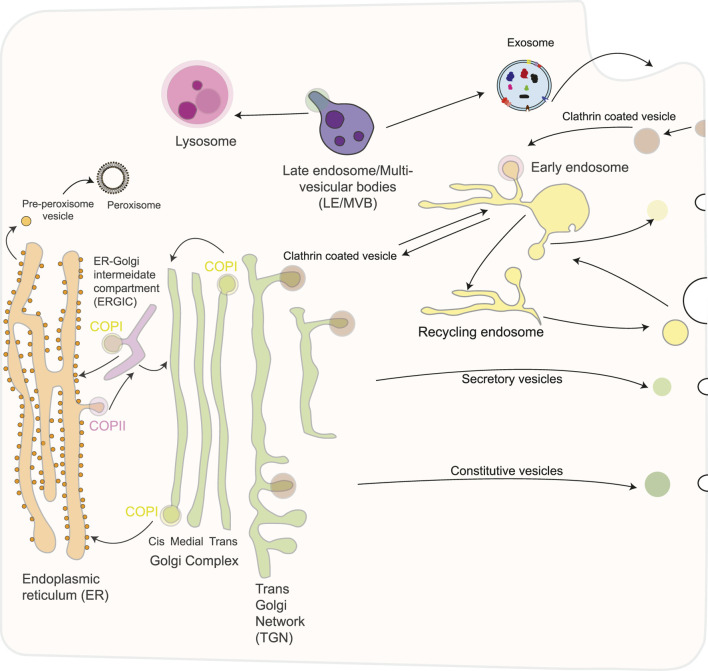
Schematic overview of the secretory pathway in eukaryotic cells. The secretory pathway consists of an exocytotic (biosynthetic) branch that begins in the endoplasmic reticulum and leads to the plasma membrane or other intracellular organelles and an endocytic limb that leads from the plasma membrane to the lysosomes. The endoplasmic reticulum exchange material with the Golgi apparatus *via* anterograde and retrograde transport. Traffic to the plasma membrane involves a constitutive and a regulated route, involving different types of transport vesicles, originating from the trans- Golgi network (TGN). Several different routes of endocytosis coexist that converge on the early endosome, including a clathrin-coated and several non-coated pathways. Furthermore, early endosomes mature into late endosomes and fuse with lysosomes, the end point of the degradative pathway. Exosomes are released after fusion of MVBs with the plasma membrane. Exosomes have a complex composition of proteins, nucleic acids, lipids, and other metabolites. Peroxisomes are generated autonomously by dividing pre-existing organelles or through a *de novo* process involving budding from the ER, followed by the importation of matrix proteins.

Cell's ability to communicate with the outside world is crucial for their survival. The plasma membrane is the interface used by cells to interact with their surroundings. Eukaryotic cells evolved a sophisticated endomembrane system (Segev et al., 2009) composed of a series of membranous organelles affecting the composition and function of the plasma membrane, among which the rough endoplasmic reticulum (RER), smooth endoplasmic reticulum (SER), Golgi apparatus (GA) and endo-lysosomes. The endocytic pathway allows the ingestion of fluids, molecules, and particles into the cell in specific locations where the plasma membrane invaginate and pinches off to form endocytic vesicles (Uzman, 2003). Endocytosis is typically a constitutive event that can also be triggered by extracellular signals. Endocytosis is a very extensive process where large regions of the plasma membrane are ingested every hour. Most of the protein and lipids endocytosed from the plasma membrane are trafficked back to the cell surface *via* exocytosis (Figure 1). The balance between endocytosis and exocytosis is crucial for processes such as signaling, nutrient uptake, and membrane repair. Extracellular vesicles also play a crucial role in cell-cell communication and trafficking.

In this review, we will provide a comprehensive overview of the role of vesicle biology in cortical development and neurodegenerative diseases.

## 2 Cellular logic of brain development

The neurons forming the brain are generated from neural stem and progenitor cells during embryonic development, in a process called neurogenesis. Neural stem and progenitors can be classified into two main classes, based on where their mitosis is taking place: apical progenitors (APs) undergo mitosis at the ventricular surface, while basal progenitors (BPs) undergo mitosis far away from the ventricle, in the SVZ, a proliferative zone found at a more basal location. Besides the location of mitosis, APs and BPs show striking cell biological differences: APs are epithelial cells with an apical and basolateral plasma membrane, while BPs typically lack an apical attachment despite often maintaining an apical-directed process and a basal attachment. The transition from AP to BP closely resembles an EMT and is commonly referred to as delamination. Several reports show that the presence and nature of progenitors' processes (of epithelial polarity or not) are regulated by polarity genes (Taverna et al., 2014). Seminal work in cells in culture clearly shows the strong interplay between polarity and trafficking. In polarized cells, the membrane is organized into apical and basolateral regions, each with its own set of proteins. Membrane trafficking plays a crucial role in ensuring that these proteins are properly directed and sorted, thus maintaining the cell's polarity (Bentley and Banker, 2016; Britt et al., 2016). In line with that concept, recent evidence has started to uncover the role of traffic and vesicle biology in brain development (Polenghi and Taverna, 2023).

## 3 Vesicles transport at the golgi apparatus

The Golgi apparatus plays a vital role in the synthesis of glycolipids, protein glycosylation, and in sorting and secretion of substances. The Golgi apparatus is composed of different regions: the cis-, medial-, and trans-Golgi network. Proteins from the endoplasmic reticulum (ER) pass through an intermediate compartment known as the ER-Golgi intermediate compartment (ERGIC), are transported to the cis Golgi, and then enter the medial and trans compartments of the Golgi stacks, where most metabolic processes occur (Figure 1) (Lowe, 2011). Modified proteins and lipids are transported to the trans-Golgi network, which acts as the central hub controlling the molecular sorting and trafficking towards the final destination, represented by, e.g., lysosomes, the plasma membrane, and the extracellular space (Mohan et al., 2023).

Proteins intended for secretion or exocytosis undergo glycosylation in the Golgi apparatus (Giraudo et al., 2001). The extent of modifications depends on several factors, like their structure and the abundance of the processing enzyme in the Golgi complex, a feature that is cell-type dependent. In addition to processing and sorting glycoproteins, the Golgi apparatus plays a critical role in lipid metabolism and sphingomyelin synthesis. Proteins, lipids, and polysaccharides are transported to their destination *via* transport vesicles that bud from the trans-Golgi network and traffic the cargo to their final destination (Cooper and Adams, 2022).

**FIGURE 2:**
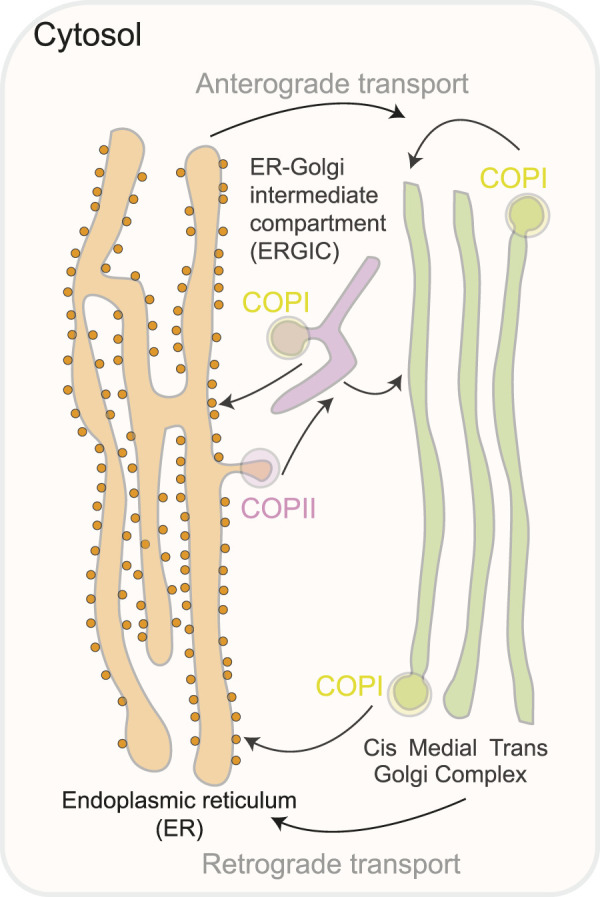
A schematic representation of the compartments involved in the transit of ER-Golgi proteins. COPII coat proteins produce vesicles that bud from the ER to start anterograde transport. The ER-Golgi intermediate compartment (ERGIC) is a pleiomorphic compartment created when COPII vesicles merge with the Golgi either immediately or while traveling there. To guarantee retrieval to the ER, COPI proteins recycle fugitive ER inhabitants and produce retrograde vesicles from the Golgi complex or the ERGIC.

Vesicles that move cargo between different organelles and between various regions of the same organelle are known as transport vesicles. The vesicle budding from a certain compartment is promoted by protein coats, which are supramolecular complexes formed on the cytosolic face of the membrane(s) that allows the vesicle to bud off and then recognise and attach to the proper target membrane compartment. There are three types of protein coats: Clathrin, Coatomer I (COPI), Coatomer II (COPII) (Buratta et al., 2020). Clathrin composes the vesicle coat in three key receptor-mediated intracellular transport pathways: the export of aggregated substances from the trans-Golgi network for regulated secretion, the delivery of lysosomal hydrolases from the trans-Golgi network to lysosomes, and receptor-mediated endocytosis at the plasma membrane (Figure 1). It is polymerized in a network that forms a polyhedral-clathrin- lattice. There are five different Adaptor protein (AP) complexes: AP-1, AP-2, and AP-3, clathrin-associated complexes, whereas AP-4 and AP-5 are not (Park and Guo, 2014). Unlike clathrin, COP proteins are not assembled in structured geometric arrangements but rather in a dense and diffuse layer (Figure 2). Each of these protein coats operates at a different stage of the endomembrane system. Here, we focus on COPI and COPII-coated vesicles that transport cargo through the ER-Golgi interface. The machinery governing the formation of coated vesicles is evolutionarily conserved (McCaughey and Stephens, 2018), and their functioning is a fundamental cellular process. In line with that, defects and mutations in these proteins impair specific transport pathways and compromise cell viability. In humans, mutations in coat proteins cause several congenital diseases collectively referred to as “coatopathies” (Table 1) (Dell'Angelica and Bonifacino, 2019).

**TABLE 1:** Summary of transport vesicles: coat proteins and associated pathologies.

Vesicle type	Coat/key protein	Trafficking/function	Intracellular/extracellular	Pathology
COPI-coated	COPI (α-COP-β′COP δCOP)	Retrograde transport (Golgi→ER, intra Golgi)	Intracellular	Microcephaly 19, primary, autosomal recessive (MCPH19), Short stature, rhizomelic, with microcephaly, micrognathia, and developmental delay (SRMMD), Alzheimer's, ALS
COPII-coated	COPII (inner SEC23-SEC24 dimers and outer SEC132- SEC312 heterotetramers)	Anterograde transport (ER→ ERGIC and Golgi)	Intracellular	Parkinson disease and SEC24b encephalopathy
Lysosomes	No coat but specific markers and enzymes (LAMPs, acid hydrolases)	Degradation of macromolecules	Intracellular	Lysosomal storage disorder (LSD), Nieman pick disease (NPC), AD, HD, PD
Peroxisomes	Pex proteins	β-oxidation of fatty acids, detoxification of ROS	Intracellular	Zellweger syndrome, X-linked adrenoleukodystrophy, AD, ALS
Exosome	Tetraspanins, CD63, CD81; regulated by depolarization, DNAJC5	Synaptic communication, neurogenesis, protein clearance, gene regulation	Extracellular	AD (amyloid, tau), PD (α-synuclein), ALS/FTLD (TDP43), ASD, Rett syndrome, Down syndrome
Microvesicles	Annexins, cytoskeletal proteins	Cell signaling, modulation of synaptic activity	Extracellular	General role in NDDs and possibly AD
Apoptotic Bodies	Caspases, phosphatidylserine	Clearance of cellular debris, inflammation regulation	Extracellular	Fetal alcohol syndrome, potential involvement in neurodevelopmental disorders
Synaptic Vesicles (SVs)	Synaptophysin, synaptotagmin, clathrin	Recycled at presynaptic terminals- relase of neurotransmitter at synapses	Intracellular	Autism (ASD), intellectual disability, epilepsy, KAND
Dense Core Vesicles (DCVs)	KIF1A (motor protein)	Soma → axons/dendrites Transport neuropeptides and hormones	Intracellular	KAND (KIF1A-associated neurological disorder), neurodegeneration
Kinesin-transported Vesicles	KIF1A, microtubules	Anterograde (cell body → synapse) Transport SVs and DCVs along axons	Intracellular	Spastic paraplegia, autism, sensory neuropathy, NESCAV syndrome
Rab-regulated Vesicles	GDI1 (Rab GDP-dissociation inhibitor)	Endosomal/synaptic membrane trafficking- Modulate vesicle fusion, memory, and signal regulation	Intracellular	Intellectual disability, memory deficits, Alzheimer's (biomarker potential)

**FIGURE 3:**
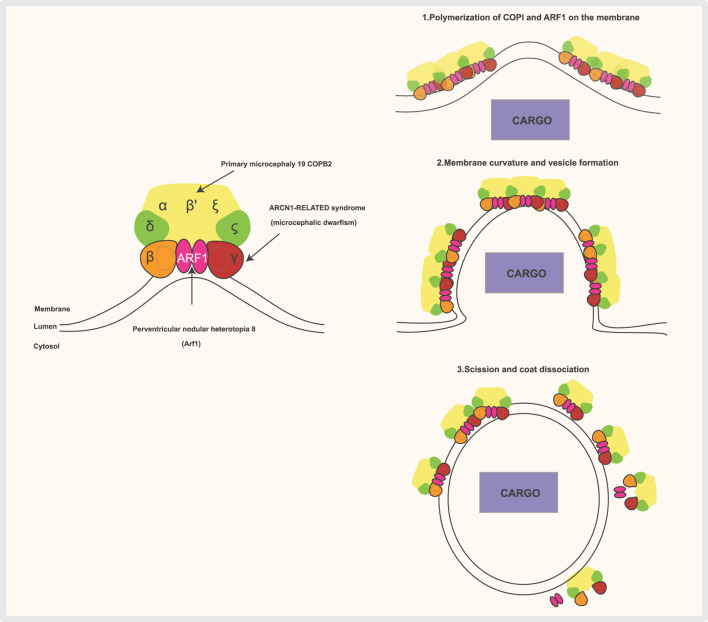
The COPI complex is composed of a scaffold “B-subcomplex” (made up of the α, β′, and ε subunits) and an adaptor “F-subcomplex” (which includes β-COP, δ-COP/Arcn1, γ-COP, and ζ-COP). Two ARF1 small GTPase molecules bind to the membrane and interact with COPI through the β-COP and γ-COP subunits when ARF1 is GTP-bound. Several subunits of the complex are associated with human diseases. COPI complexes and ARF1 form triads, with cargo (such as ER-resident proteins requiring retrieval from the Golgi) selected by direct interaction with COPI subunits or through transmembrane receptors that bind COPI. COPI then polymerizes on the membrane, inducing deformation and curvature, which leads to the budding and scission of the transport vesicle. Once the vesicle is released, the coat is shed, and ARF1 and COPI dissociate.

Anterograde transport moves proteins and lipids from the endoplasmic reticulum (ER) to the Golgi apparatus for modification and sorting. In contrast, retrograde transport carries enzymes and other molecules from the Golgi back to the ER to support proper cellular function and organization. For the anterograde transport (ERàGA), the cargo exit at the ER-exit sites (ERES) is mediated by COPII coat (Figure 2), which binds and concentrates the cargo into vesicles. COPII recruitment is mediated by an active GTP-bound form of small GTPase Sar1. Membrane deformation that leads to the formation of COPII vesicles occurs *via* Sar1 and Sec23/24 complex and is stabilized by Sec 13/31 complex Sar1 can deform membranes *in vitro*, which suggests it is ability to facilitate accurate vesicle scission, similar to dynamin, which mediates the scission of clathrin-coated vesicles On the other hand, for retrograde transport (GAàER and intra-GA) the cargo exit in multiple stages of ER-to- Golgi transport depends on COPI (Shima et al., 1999; Spang and Schekman, 1998). COPI is formed of seven subunits (α, β, β′, γ, δ, ε and ζ) (Figure 3) (Arakel and Schwappach, 2018). ARF1, a small GTPase, catalyzes COPI recruitment to the vesicle. In its GTP-bound form, ARF1 becomes stabilized in the membrane and initiates recruitment of COPI through binding with the trunk domains of the β-COP and γ-COP subunits (Figure 3) (Yu et al., 2012). COPI causes increased curvature of the membrane, forming a vesicle, and then scission occurs. After the vesicle is released, the COPI coat is shed, and ARF1 and COPI can dissociate (Figure 3). The main functions of COPI are trafficking ER-proteins back from the Golgi to the ER, recycling certain transmembrane cargo receptors from the Cis*-*Golgi and ERGIC, and transporting intra-Golgi retrograde cargo. Moreover, COPI is involved in lipid droplet formation and lipolysis. Depletion of β-COP results in defects in cargo transport, compartmentalization of the ERGIC trans-Golgi network, Golgi, and recycling endosomes (Macken et al., 2021). COPII is formed of an inner layer of SEC23-SEC24 dimers and an outer layer of SEC132-SEC312 heterotetramers, which are sequentially recruited to create a flexible cage around vesicles (Zanetti et al., 2013). COPII functions as a coat for virtually all proteins that exit the ER towards the ERGIC and Golgi complex, the mechanisms of cargo recognition by COPII are necessarily quite diverse (McCaughey and Stephens, 2018).

Conventionally, most eukaryotic secretory proteins are translocated to the ER (led by an amino-terminal signal peptide (leader sequence) and transported to the cell surface through vesicles that pass through the GA. However, discoveries have shed light on additional secretory pathway(s), called unconventional secretory pathway(s). Cells use this unconventional secretory pathway to secrete many proteins that lack a signal sequence (leaderless proteins) (Filaquier et al., 2022; Malhotra, 2013; Rabouille, 2017; Zhang and Schekman, 2013). Although COPII-coated vesicles are essential for exporting secretory cargo from the ER, they are still too small to transport large molecules and bulky cargo, such as collagens, mucins, and lipoprotein particles. The transport of bulky cargo is mediated by tunnels. Once a tunnel is filled with cargo, its connection to the ER exit site (ERES) is severed. It remains unclear whether this cargo-filled saccule fuses with the cis-Golgi cisterna or matures until it consists solely of cargo destined for secretion. In most cell types, both types of exit sites are utilized: small cargoes are exported *via* COPII vesicles. In contrast, large cargoes like collagens are transported *via* tunnels—these tunnels are devoided of COPII components. COPII components were only found to localize to the neck of these tubules, implicating that the central role of COPII is to concentrate cargo into carriers (Malhotra, 2025; Phuyal and Farhan, 2021; Zeuschner et al., 2006). The unconventional secretory pathway(s) has also been shown to operate in solid forms generated from different types of organelles: fractions of lysosomes and autophagosomes (APh) undergo exocytosis and also exosomes and ectosomes, with their extracellular vesicles (EVs) (Jacopo, 2023) (Figure 1).

### 3.1 Coatomers in brain development

Proper regulation of vesicle function and location is required across the full spectrum of eukaryotic cell biology, from energy generation at the mitochondrion to the regulation of gene expression in the nucleus. This is especially true of the cells of the central nervous system, such as neurons and glial cells. The human brain is considered the body's most complex organ, and this could make it more vulnerable to subtle abnormalities. The human neocortex is a novelly evolved structure of the human brain; it is responsible for higher-order cognitive functions. The development of the cerebral cortex is a precise process that is orchestrated to produce its six-layered laminar structure (Florio and Huttner, 2014). During cortical development, apical radial glia residing in the ventricular zone (VZ) will produce neurons directly (direct neurogenesis) or indirectly *via* intermediate progenitors (IPs). IPs divide symmetrically to produce neurons or asymmetrically to amplify their progenitor pool. Later in development, APs give rise to basal radial glia (bRGs), which lack attachment to the apical surface of the ventricular zone. bRGs possess a higher proliferative capacity that is considered one of the factors responsible for human cerebral cortex folding. Of note, APs and bRGs show remarkable cell biological and functional polarization, a feature that might make them more vulnerable to subtle changes in the trafficking and sorting machinery (Taverna et al., 2014; 2016). An example in that respect is shown by an elegant study based on subcellular live imaging of the mouse developing brain, in which the Baffet group showed that Rab6 post-Golgi vesicles are crucial for the maintenance of the integrity of the neuroepithelium. Impairment of the Rab6/dynein/Lis pathway leads to an impairment of apical junctions' integrity, causing APs delamination and leading to the formation of a BP (Brault et al., 2022). Of note, these data also suggest an interplay between the trafficking machinery and fate specification.

Mutations in COPB1 that result in the altered interaction between β-COPI and β′-COPI were found in patients suffering from severe cataracts, developmental delay, and microcephaly, a clinical manifestation often associated with a change in the mode of division of neural stem cells. Indels into genomic copb1 in *Xenopus tropicalis* recapitulated the clinical manifestations found in patients (Macken et al., 2021). RNA interference experiments showed that COPB1 plays a critical role in cell division, thus providing a mechanistic link to the microcephaly phenotype in COPB1 patients (Kittler et al., 2007) (Table 1).

Similarly, COPB2 gene encoding β^'^-COPI affects brain development: two siblings suffering from microcephaly, cortical blindness, and developmental delay were found to share a large span (16.8 Mega bases) of homozygosity in chromosome 3, including a rare missense variant in *COPB2*. Moreover, neurospheres derived from animals with Copb2 variants grew less than control. These results indicate the general requirement for COPB2 in embryogenesis and a specific role in corticogenesis (DiStasio et al., 2017).

Although CRISPR-Cas9 generated mice carrying the same patients' missense variant COPB2 (p-R254C) appeared to be normal, heterozygous mice carrying the p. R254C substitution over a null allele showed smaller bodies and brain sizes and a reduced cortical area, with a specific decrease in CTIP2-positive, layer V neurons. A possible explanation for this observation is that the COPB2 p. R254C variant acts as a hypomorphic allele and that human brain development is more sensitive to the loss of COPI function than the mouse brain. The increased sensitivity of the human brain to trafficking defects might be explained by the fact that human progenitors, especially APs and bRGs, are way longer than their mouse counterpart, making them more relying on a very tight and precise regulation of COPI vesicular trafficking to reach distant locations (Dell'Angelica and Bonifacino, 2019; Martínez-Menárguez et al., 2019).

The SEC24B gene encoding a component of COPII vesicle machinery, has been associated with neurodevelopmental disorders. In a study of 163 stillborn or miscarried fetuses with neural tube defects monoallelic missense variants in SEC24B were found in four cases (Yang et al., 2013). Another case-control sequencing study suggested an increased number of potentially damaging mutations in the SEC24B gene among patients with mesial temporal lobe epilepsy with hippocampal sclerosis (Dell'Angelica and Bonifacino, 2019). Although suggestive, Future work is required to confirm the potential role of SEC24B mutations in brain development.

Monoallelic germline loss-of-function mutations in the gene encoding δ-COP (*ARCN1*; the coatomer subunit delta of COPI) were found in four patients with a history of intrauterine growth retardation. These patients showed short stature with disproportionally small proximal limbs (rhizomelia), microcephaly, micrognathia (small jaw), and mild developmental delay (Izumi et al., 2016). Mice with a homozygous missense mutation in *Arcn1* showed several similarities to the individuals with *ARCN1*-related syndrome, such as low body weight and neurological phenotype, including ataxia due to cerebellar degeneration (Xu et al., 2010). *ARCN1* mutant cell lines show endoplasmic reticulum stress, suggesting the involvement of ER stress response in the pathogenesis of this disorder (Ishikawa et al., 2013; X; Zhang et al., 2007).

Interestingly, ER stress was reported to regulate brain development (Laguesse et al., 2015), suggesting a possible mechanistic link between defective ARCN1 and impaired brain development.

### 3.2 Coatomers in neurodegeneration

Neurodegenerative diseases—such as Alzheimer's, Parkinson's, and ALS—are complex pathological conditions that compromise various subcellular compartments and organelles and affect a range of biological processes, such as protein maturation, mitochondrial function, autophagy, and protein trafficking (Haass et al., 2012).

One of the distinctive features of Alzheimer's disease is the presence of amyloid plaques composed of aggregated amyloid b (Ab) peptides that result from the sequential cleavage of the amyloid precursor protein (APP). Recent data suggest that APP processing might be affected by its trajectory through the early secretory pathway involving the COP complexes that regulate retrograde and anterograde transport (Choy et al., 2012) (Table 1). Manipulating COPI by δ-COP subunit KO results in an accumulation of APP in the Golgi and a parallel decrease in its maturation and expression at the plasma membrane, leading to a reduced production of Aβ peptides.

These data shed a new light on AD studies, pointing to an involvement of trafficking and maturation of APP and not only on the accessibility and efficiency of the proteases that cleave APP (Bettayeb et al., 2016).

It has been shown that the accumulation of α-synuclein (α-syn), a hallmark of Parkinson's disease (PD), inhibits the Unfolded Protein Response (UPR) pathway, particularly the ATF6 branch, which is crucial for cell survival. Studies showed that this inhibition is achieved by α-syn interfering with ATF6 processing and its incorporation into COPII vesicles, which are critical for transport to the Golgi apparatus. This disruption leads to impaired ER-associated degradation (ERAD) and increased pro-apoptotic signaling, contributing to the neurodegeneration associated with PD (Credle et al., 2015).

Amyotrophic lateral sclerosis (ALS) is a neurodegenerative disorder mainly affecting upper and lower motor neurons. ALS was among the first neurodegenerative diseases where Golgi fragmentation was described, an observation consistently found across all patients and animal models linked to mutations in SOD1, TARDBP (TDP-43), VAPB, and C9Orf72 (Gonatas et al., 1992; Mourelatos et al., 1990). A study performed on mice mutated in the TBCE gene, which encodes the cis-Golgi localized tubulin-binding cofactor E, showed that these mutations caused alterations in Golgi microtubules, impeding the maintenance of the Golgi architecture. This fragmentation occurs as a result of the downregulation of COPI coat components, the dispersion of Golgi tethers, and a significant accumulation of ER-Golgi SNAREs (Soluble N-ethylmaleimide-sensitive factor Attachment Protein Receptors), which are proteins involved in vesicle fusion with target membranes during processes such as exocytosis and endocytosis. This study proposed that defects in COPI vesicles, microtubules, and their interaction may also underlie Golgi fragmentation in human ALS patients (Schaefer et al., 2007). Taken together, these data also suggest that alterations in intracellular traffic might be a cause, rather than a consequence, of neurodegenerative diseases.

The function(s) of COPI in neurons is not limited to regulating intracellular membrane traffic. Indeed, it has been shown that RNAs are associated with COPI in neuronal cells. These results suggest that COPa complexes incorporate a specific set of RNAs that harbor putative neurite-targeting motifs and display significant overlap with neuronal RNA-binding proteins (RBPs) known to localize to the plasma membrane and cytoskeleton (Todd et al., 2013). These data are intriguing, as they suggest a multi-level regulation of intracellular traffic by COPI.

## 4 Lysosomes

**FIGURE 4:**
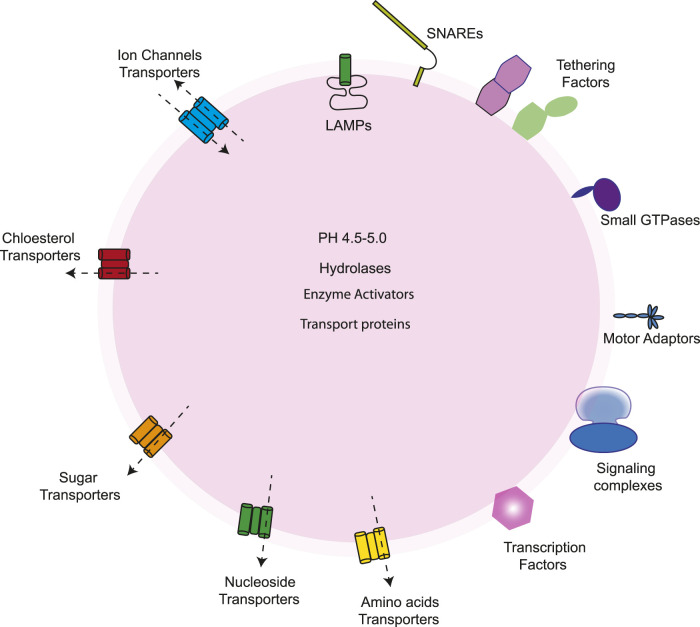
The lysosome contains a variety of proteins, including those found in the lumen, integral membrane proteins, and peripheral proteins. Its lumen holds acid hydrolases for substrate degradation, enzyme activators, protective factors, and transport proteins like NPC2, which moves cholesterol to NPC1 for export. V-ATPase regulates the acidic pH in the membrane. The membrane also has glycosylated lysosome-associated membrane proteins (LAMPs) that protect the membrane, along with ion channels, lipid transporters, solute carriers for exporting sugars, amino acids, and other degraded products, and SNARE proteins that facilitate fusion with other organelles. On the cytosolic side, lysosomes interact with various proteins, including mTORC1 and its regulators, transcription factors such as TFEB and TFE3, factors aiding fusion with other organelles, scaffold complexes connecting lysosomes to microtubule motors, and small GTPases that manage the recruitment and activation of these components.

The lysosome is considered the “garbage disposal system of the cell,” and it is responsible for the degradation of a variety of biological macromolecules, including proteins, lipids, carbohydrates, and nucleic acids. There are various routes by which macromolecules reach the lysosomes, including the endocytic and phagocytic pathways (Figure 1). The lysosomal lumen contains more than 60 acid hydrolases that degrade macromolecules and make them available to be reutilized by other metabolic processes (Figure 4). Lysosomes are distributed throughout the cytoplasm, and their number in a mammalian cell varies from 50 to 1000 (Ballabio and Bonifacino, 2020; Saftig and Klumperman, 2009). Recently, there has been growing evidence of the role of lysosomal dysfunction in human diseases (Table 1).

### 4.1 Lysosomes in brain development

The balance of differentiation, proliferation, and cell death rates in the developing brain are critical for neurogenesis in the developing brain (Fernández et al., 2016; Taverna et al., 2014). A recent study showcased the role of lysosomes in mammalian cortical development, showing that during asymmetric cell division giving rise to an RGC and an IP, the RGC daughter cell tends to inherit most of the lysosomes present in the mother cells, while the differentiating daughter cell relies mainly on the *de novo* biosynthesis of lysosomes (Zou et al., 2024). These data are intriguing, as one can postulate the presence of an organelle senescence mechanism linked to a cell's self-renewing or differentiation potential.

There are emerging data about the mechanisms linking lysosomal defects to disease. Among these diseases is lysosomal storage disorder (LSD), a rare monogenic inherited disease characterized by a progressive, multi-systemic phenotype often associated with early-onset neurodegeneration. LSD manifestations include dysmorphic features, musculoskeletal abnormalities, organomegaly, seizures, hypotonia, ataxia, progressive cognitive and motor retardation, and hydrops fetalis in severe cases (Kingma et al., 2015). LSD is mainly characterized by mutations in lysosomal proteins or proteins nonrelated to the lysosome that eventually lead to the impairment of lysosome-mediated degradation processes and accumulation of undegraded substrate (Parenti et al., 2015; Platt et al., 2018).

Niemann-Pick Type-C disease (NPC) is considered one of the LSDs; it is characterized by neurovisceral accumulation of lipids that leads to neurodegeneration, ataxia, dementia, and death. NPC is often present in children between middle and late childhood. Usually, it appears with early symptoms such as clumsiness and vertical gaze palsy, eventually leading to ataxia (Mengel et al., 2013). On the other hand, adult onset of NPC has been associated with mental disorders such as schizophrenia, attention deficit disorder (ADD), and depression. NPC1, the protein mutated in NPC, is a lysosomal and an endosomal protein that transports cholesterol out of these organelles in the secretory pathway (Scott and Ioannou, 2004). NPC particularly affects Purkinje cells (PC) in the cerebellum. Npc1^−/−^ mice microglia accumulate phagosome and auto-fluorescent material and attack PCs, leading to their degeneration. Moreover, in Npc1^−/−^ mice, brain degeneration is widely distributed. It can be observed as early as 3 weeks postnatally in the lateral geniculate nuclei (LGNd) and ventral posterior medial (VPM) thalamic nuclei, and by the age of 10 weeks, most PCs disappear (Boyle et al., 2020; Caporali et al., 2016; Pressey et al., 2012; Rava et al., 2022). A recent study using Npc1 late-onset mouse mutant model showed that developing dendrites in the cerebellum are significantly deficient in mitochondria. Nevertheless, they also found that anabolic (mTORC1 - mechanistic Target of Rapamycin Complex 1) and catabolic (TFEB - Transcription Factor EB) signaling pathways were not only disrupted but simultaneously activated in NPC1-deficient PCs, suggesting a loss of metabolic balance (Kim et al., 2023). It would be interesting to understand the role of metabolic and secretory pathways and their interplay in neurodevelopment since many mutations associated with mTORC1 regulation (e.g., Pten, Tsc1, Tsc2, Pi3k) lead to neurodevelopmental disorders like autism spectrum disorder (Ganesan et al., 2019).

### 4.2 Lysosomes in neurodegeneration

Lysosomal dysfunction has been reported in several neurodegenerative diseases, such as (Parkinson's disease (PD), Alzheimer's disease (AD), Huntington's disease (HD), amyotrophic lateral sclerosis (ALS), dementia with Lewy bodies, and Charcot–Marie–Tooth disease, the most common neuromuscular disorder (Ballabio and Bonifacino, 2020; Nixon, 2013). AD patients carrying the mutation in the presenilin one gene (PSEN1) exhibit lysosomal and autophagic dysfunction, defects in lysosome acidification, and Ca^+2^ homeostasis (Coen et al., 2012; Lee J. H. et al., 2015). Of note, a substantial fraction of PD patients carries mutations in lysosomal and autophagic genes. Heterozygous mutation of lysosomal enzyme β-glucocerebrosidase (GBA) gene, whose homozygous mutations cause the LSD Gaucher disease, is a major predisposing factor in Parkinson's disease. Gaucher disease is characterized by the accumulation of lysosomal glucosylceramide- GBA substrate- (GluCer), along with the accumulation of misfolded mutant GBA in the ER. Previous data demonstrated that chronic pharmacological inhibition of GBA using the selective inhibitor for lysosomal GCase, conduritol-b-epoxide (CBE), promoted the accumulation of a-synuclein aggregates and neuronal cell death by disrupting lysosomes and inducing global neuroinflammation in mice (Rocha et al., 2015). Furthermore, disruption of GBA in primary culture and human iPSCs leads to the accumulation of a-synuclein, in addition to Glucer acting as a stabilizer of a-synuclein, where the accumulation of a-synuclein itself inhibits GBA lysosomal function, pointing to a self-propagating feedback-loop in PD (Mazzulli et al., 2011). Lysosomal exocytosis is an unconventional secretion pathways; it is an ubiquitous Ca^2+^ dependent mechanism that plays a crucial role in several physiological processes, such as plasma membrane repair, bone resorption, melanocyte function, immune response, and antigen presentation (Buratta et al., 2020; Filaquier et al., 2022). In a recent study based on compound screens in a zebrafish model of neurodegenerative disorders and tauopathies, the inhibition of carbonic anhydrase (CA) was found to increase tau protein elimination in neurons, in a proteasomal and autophagic degradation-independent manner. Inhibiting CA in the SH-SY5Y expressing mutant tau-P301L reduces intracellular tau by increasing its secretion to the extracellular medium. The associated increase of the lysosomal cathepsin D in the extracellular medium and the redistribution of lysosomes to the cell periphery after CA inhibition suggest that tau is secreted through lysosomal exocytosis, a pathway previously associated with cellular clearance (Lopez et al., 2024). Moreover, the changes in the lysosomal redistribution induced by CA inhibition were accompanied by changes in the lysosomal pH, previously shown to promote lysosomal exocytosis.

Intriguingly, in Down syndrome (trisomy 21), a neurodevelopmental disorder that predisposes the early onset of AD, the extra copy of amyloid precursor gene (APP) on chromosome 21 leads to the accumulation of β-cleaved carboxy-terminal fragment of APP (APP-β-CTF or C99), in turn impairing of lysosomal acidification *via* the inhibition of v-ATPase (Jiang et al., 2019).

The lysosome is an organelle that holds as a key place at the crossroads of multiple intracellular trafficking pathways, both conventional and unconventional (Jacopo, 2023). In line with that, data obtained so far suggest that improved therapeutic targeting of lysosomes may represent a promising therapy to rescue the neurodegenerative disease phenotype.

## 5 Extracellular vesicles

Cell-cell communication occurs *via* direct contact or by secretion of soluble factors. Cells can interact and influence the extracellular environment and other cells also by releasing extracellular vesicles (EVs) (Figure 1), which have several functions depending on their origin and molecular makeup (Raposo and Stoorvogel, 2013).

Conceptually, EV release could be seen from two different points of view. It is either a mechanism to eliminate molecules/organelles from a cell, therefore influencing the molecular makeup of the releasing cells, or a mechanism that carries molecules/organelles to neighboring cells, influencing their molecular composition or state. Intriguingly, the two scenarios are not mutually exclusive, suggesting the existence of a complex and combinatorial cell-cell communication system acting both in cis and trans.

**FIGURE 5:**
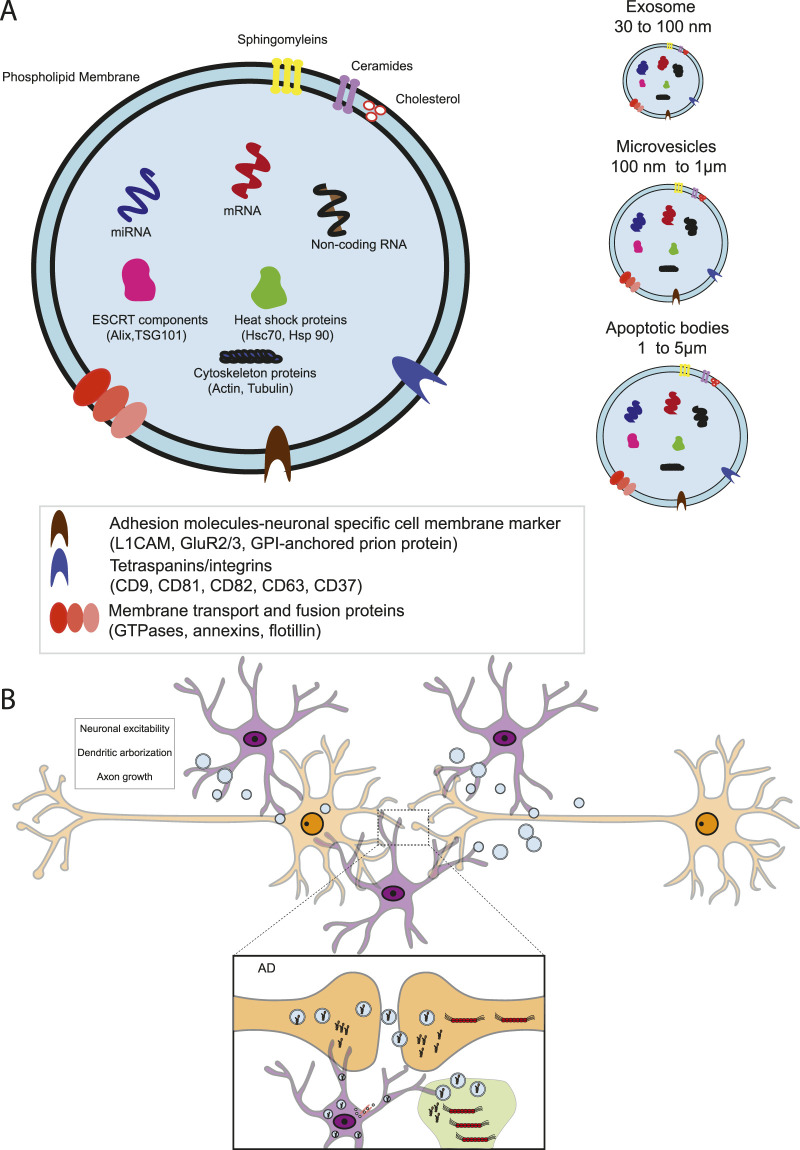
Schematic depiction of the extracellular vesicles. **(A)** EVs include exosomes, microvesicles, and apoptotic bodies. The outer EV membrane contains lipid particles and transmembrane proteins that are altered in response to environmental exposures. EVs also contain biologically active cargo, including proteins, microRNAs, tRNA, mRNAs, and polynucleotides. The cellular origin of EVs will define the cargo content and signaling capacity. For example, neural-derived exosomes carry synaptic cell adhesion molecules: neuronal-specific cell membrane marker L1CAM, GPI-anchored prion protein, and GluR2/3. Proteins involved in vesicular trafficking, such as Rab proteins, annexins, and cytoskeletal proteins, are present in EVs derived from neuronal and non-neuronal cells. **(B)** intercellular communications of CNS cells *via* EVs. EV-mediated protein and nucleic acid transfer must occur between neurons, astroglia, microglia, and oligodendrocytes. Astroglia can secrete large EVs and small microvesicles (represented by pale blue vesicles) to modulate neuronal development and activity. In Alzheimer's disease (AD), extracellular and intercellular trafficking of microglia EVs carrying misfolded Aβ and tau proteins across the synapse.

From a structural point of view, EVs are delimited by a lipid membrane bilayer and are actively secreted by almost all cells. The membrane protects EVs cargo from destruction by the extracellular environment (Figure 5A) (Kalluri and LeBleu, 2020). Based on their dimension, composition, and origin, EVs are classified into exosomes, microvesicles, and apoptotic bodies. Exosomes are generally small, ranging from 30 to 200 nm, and are released by the fusion of multivesicular bodies with the plasma membrane; on the other hand, microvesicles (MVs), whose sizes vary between 100 and 1000 nm, are directly released from the outward blebbing of the plasma membrane. The largest class of EVs is represented by apoptotic bodies (1–5 mm in diameter), which are shed from cells undergoing apoptosis (Figure 5A) (Gomes et al., 2020).

The heterogeneity of EVs mainly depends on their cargo: nucleic acids (RNAs encapsulated in EVs mainly include non-coding RNAs such as mRNAs, long non-coding RNAs (lncRNAs), circular RNAs, and micro RNAs), proteins, lipids, cytokines, chemokines, and the end-stage neurotoxic and pathogenic metabolic products (Kalluri and LeBleu, 2020).

### 5.1 EVs in brain development

EVs influence the extracellular environment composition, and several studies showed their role in brain development. Marzesco et al. reported that during embryonic mouse brain development, neuroepithelial stem cells release EVs that carry the stem cell marker prominin-1 (CD133) in the lumen of the neural tube (Marzesco et al., 2005). It was later found that these particles are derived from the primary cilia or from the complete abscission of the midbody (Dubreuil et al., 2007). Interestingly, proliferating and self-renewing neural stem cells were found to differ regarding the release or inheritance of the midbody (Ettinger et al., 2011). These data suggest that neural stem cells' proliferation/differentiation balance might be influenced (or at least paralleled) by different competencies in EV release. EVs released in human and mouse retinal organoids contain molecular cargo associated with post-translational modification and regulation of human retinal development (Zhou et al., 2018; 2021).

It has been shown that cortical and hippocampal neurons *in vitro* release exosomes, suggesting the role of EVs in mediating synaptic transmission and neuronal communication *via* AMPA and NMDA receptors (Lachenal et al., 2011). Moreover, neuronal and astrocyte culture *in vitro* release exosomes carrying Lcam1 and specific units of glutamate receptors (GPI anchored prion protein and the GluR2/3 but not the NR1 subunits of glutamate receptors). This release of exosomes is regulated by depolarization and, therefore, regulated concomitantly with neuronal activity and SV release (Fauré et al., 2006).

A recent study using human-induced pluripotent stem cell (iPSC)-derived neural cells in 2D and 3D (brain organoids) showed EVs' heterogeneity and how it changes during developmental time points. EVs from brain organoids were enriched at d15 in proteins regulating cell cycle and RNA splicing, at day 40 in intracellular transport and mitochondrial membrane, at day 200 in ribosome biogenesis and mitochondrial proteins, and at the latest time point (day 360) in proteins regulating locomotion, secretion, and cell motility, suggesting a time-dependent mechanism for the release and disposal of cellular components. It would be interesting to understand if the disposal of cellular elements is associated with precise steps of cell maturation and associated changes in the overall cell's molecular makeup. Moreover, the authors showed that EVs transporting YAP1-transcription factor in neural progenitor cells are transported to the nucleus of recipient progenitor cells during mitosis and are then localized to the nucleus of the daughter cell. This data suggests that the role of EVs in cell-cell communication might extend to transcriptional regulation (Forero et al., 2024).

In line with their multiple roles in maintaining neurons and forming synapses, alterations in EV biology have been described in neurodevelopmental disorders such as autism spectrum, Rett syndrome, Down syndrome, and fetal alcohol syndrome (see Table 2).

**TABLE 2:** Extracellular vesicle cargo and its implication in neurodevelopmental disorders.

Disease	Manifestations	EVs cargo	Ref.
Rett Syndrome	Severe neurological disorder due to mutations in the gene encoding the methyl-CpG-binding protein 2 (MeCP2), localized in the X chromosome. Rett syndrome leads to developmental regression with symptoms ranging from loss of speech, acquired movement skills and severe cognitive impairment after an apparent normal development.	Proteome analysis of exosomes from hiPSC-derived neurons from MeCP2 LOF-disesase cells and isogenic controls revealed a downregulation in signaling proteins associated with neuronal maturation, axon guidance and synaptogenesis.	Marchetto et al. (2010), Sharma et al. (2019)
Autism spectrum	A lifelong neurodevelopmental pathology with core abnormalities in sociability and communication capacity. Stereotyped behaviors and interests. Inflammation in the CNS and neuro-immune crosstalk dysregulations are prevalent in ASD patients, thus affecting the activity and proliferation of glial cells, astrocytes, and microglia.	EVs isolated from ASD children's serum (i) show an increase in total protein concentration and in the amount of mtDNA and (ii) ASD stimulated cultured human microglial cells to secrete more pro-inflammatory cytokines, such as IL-1β.	Matta et al. (2019), Pascual et al. (2020), Tsilioni and Theoharides (2018)
Down syndrome	Human genetic disease caused by trisomy of chromosome 21 (Hsa21). Characterized by early developmental brain abnormalities and early onset of Alzheimer's disease (AD).	-Exosomes from blood samples from DS patients showed an Increased CD81 levels (more abundant neuronal exosomes secreted). -Neuronal exosomes contained Aβ peptide products and hyper-phosphorylated Tau (P-Tau).	Hamlett et al. (2015), Hamlett et al., 2017, Hamlett et al., (2018), Holtzman et al. (1996), Milenkovic et al. (2018)
Alcohol fetal syndrome	Prenatal exposure to alcohol can cause developmental deficits, termed fetal alcohol spectrum disorders (FASDs), which include growth deficits and neurodevelopmental delay, affecting cognition and behavior.	Cultured neurons and astrocytes showed an increase in number of EVs upon ethanol exposure, with higher content of inflammatory-related proteins, such as TLR4, NF B-p65, IL-1R, caspase-1 and NLRP3, as well as miRNAs (miR-146a, miR-182 and miR-200b.	Ibáñez et al. (2019), Mahnke et al. (2019), Moore et al. (2014)

### 5.2 EVs in neurodegeneration

Since the first observation that exosomes accumulate in amyloid plaques of AD patients (Jacopo, 2023), there is a growing interest in the role of EVs in neurodegenerative diseases. Exosome secretion (a type of unconventional protein secretion) is thought to be a method of getting rid of misfolded and aggregated proteins; their transfer to other neighboring cells, however, might end up propagating the disease even further; this observation was confirmed *in vitro* and *in vivo*, showing that exosomes from neuronal cells indeed contain amyloid proteins precursors (Pluta et al., 2018; Rajendran et al., 2006). The possible involvement of exosomes in AD was confirmed by the finding of hyperphosphorylated tau protein in exosomes from human CSF (Pluta et al., 2018). Tau protein accumulation in AD patients could be caused by microglia, which may participate in spreading the tau protein *via* exosomes (Figure 5B) (De Toro et al., 2015). Moreover, hyperphosphorylated tau protein in exosomes in transgenic mice could be a sign that post-translational modification is a requirement for the spreading of tau proteins to other cells *via* exosomes (Fiandaca et al., 2015; Saman et al., 2012; Wei et al., 2017). Taken together, the data indicate that exosomes could exacerbate tauopathies and cognitive loss in AD patients. Exosomes could potentially be used as a biomarker for AD, becoming a valuable diagnostic tool for early AD screening before the onset (Cai et al., 2018). Exosomes carrying transactive response binding protein-43 (TDP43) are also markers of amyotrophic lateral sclerosis and frontotemporal lobar degeneration (Iguchi et al., 2016; Soo et al., 2015). In PD, exosomes show a similar pattern where a synuclein is secreted *via* exosomes and transferred in a calcium-dependent manner to healthy cells -mainly neurons and astrocytes-where they exert toxic effects causing the death of the recipient cells (Danzer et al., 2012; Emmanouilidou et al., 2010).

DNAJC5, also known as cysteine string protein α (CSPα), is a co-chaperone of HSC70; it controls the extracellular release of many neurodegenerative disease proteins (Abela et al., 2024; Fontaine et al., 2016). It has been shown to control the release of neurodegenerative disease proteins, but the mechanism of action of this protein in unconventional secretion remains elusive. A recent study showed that DNAJC5-regulated α-syn secretion is different in different *in vitro* models, and proposed that palmitoylated DNAJC5 oligomers function at a step involving membrane translocation of cytosolic α-syn, enabling it to become competent for secretion. This was accompanied by the localization of DNAJC5 and α-syn within enlarged endosomes, in preparation for their secretion into the extracellular space (Wu et al., 2023).

Recently, EV biology has been exploited to treat neurodegenerative diseases because of their ability to cross the blood-brain barrier (BBB) (Saeedi et al., 2019). Recent reports in AD showed that stem cell-derived EVs have neuroprotective and immunomodulatory properties (Garcia-Contreras and Thakor, 2023). Studies on the application of EVs in the treatment of NDD are scarce. Recent work shows that exosomes isolated from adipose MSC stem cells and administered intranasally to a mouse model of ASD (Shank3, presenting autistic-like behaviors and ASD symptoms) led to an improvement in behavioral phenotype (Geffen et al., 2020; Perets et al., 2018). It is worth noting that before developing any clinical application that uses EVs, the cargo loading and mechanism of action should be carefully studied, especially since EVs isolated from MSCs could show variations depending on the different cell sources (de Almeida Fuzeta et al., 2020).

## 6 Peroxisomes

**FIGURE 6:**
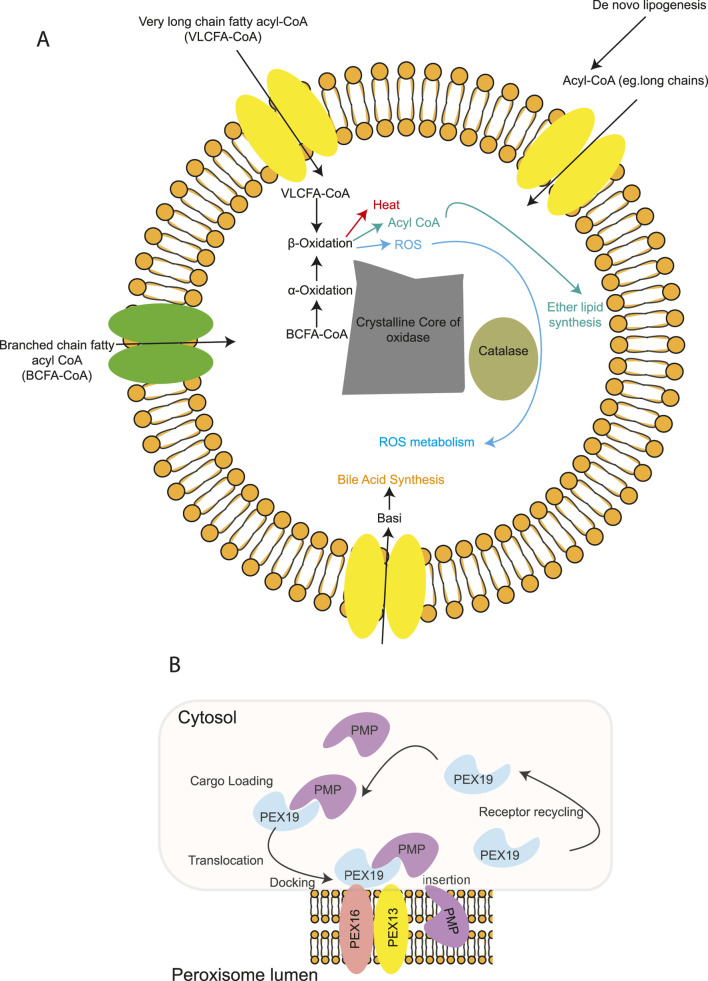
Schematic illustration of the peroxisome and how peroxisomal membrane import occurs. **(A)** Peroxisome structures are single-membrane structures formed of lipid bilayer-bound vesicles involved in energy metabolism and lipid biosynthesis. The main metabolic functions of peroxisomes in mammalian cells include β-oxidation of very long-chain fatty acids, α-oxidation of branched-chain fatty acids, synthesis of bile acids and ether-linked phospholipids, and removal of reactive oxygen species. Peroxisomes in many, but not all, cell types contain a dense crystalline core of oxidative enzymes. **(B)** Peroxisomal membrane proteins (PMPs) are imported post-translationally to the peroxisomal membrane. Pex19 is a soluble chaperone that binds to PMPs and transports them to the peroxisomal membrane, where it docks with a complex containing Pex16 and Pex3. Pex19 is recycled back to the cytosol following this interaction with the PMP.

Peroxisomes are small (0.2–1 µm in diameter), single membrane-bound organelles found in the cytoplasm, separating their internal contents from the rest of the cell (Figure 6A). They are highly dynamic, ubiquitous organelles whose number, shape, and cargo are variable and depend not only on the tissue but also on changes in the extracellular environment. Peroxisomes are biogenetically linked to the endoplasmic reticulum. Peroxisomes rely on proteins called **peroxins**, encoded by **Pex genes**, for their biogenesis, division, and inheritance. A subset of peroxins localize to or integrate into the ER, where they accumulate in specialized subdomains. These regions undergo a membrane scission event, releasing pre-peroxisomal vesicles that mature into fully functional peroxisomes (Figure 1). Over 30 peroxins have been identified in yeast, making it a model organism due to its genetic accessibility and inducible peroxisome proliferation. Many peroxins are conserved in mammals and function in membrane assembly, protein import *via* peroxisomal targeting sequences, and docking of peroxisomal proteins (Figure 6B) (Lodhi and Semenkovich, 2014).

Peroxisomes play critical roles in catabolic and anabolic processes; they are essential for metabolic processes such as lipid metabolism, particularly fatty acid b oxidation, myelin sheath lipid metabolism, and metabolism of reactive oxygen species (ROS) (Hiltunen et al., 2003; Islinger et al., 2018). In line with that, dysfunction in peroxisomes is linked with various human disorders, including peroxisome biogenesis disorders (PBDs) and peroxisomal enzyme deficiencies (PEDs) (Table 1) (Braverman et al., 2013).

Peroxisomes are abundant in all cell types and exert specific functions in the CNS. They have been detected in all cell types present in the brain: neurons, oligodendrocytes, astrocytes, microglia, and endothelial cells. They appear as electron-dense single membrane organelles, and brain peroxisomes—specifically neuronal peroxisomes—are smaller than those found in other tissues (Arnold et al., 1979).

Peroxisomes participate in the biosynthesis of complex lipids such as ether phospholipids, an important component of myelin that ensheathes oligodendrocytes (Bazinet and Layé, 2014). Studies on the peripheral nerves in mice showed that peroxisomes are diffusely distributed in the myelin sheath of Schwann cells at early stages. However, in later stages, peroxisomes are enriched at the myelin loops of the paranodal region. This region serves as an axonal-glial contact site that flanks the node of Ranvier, a substructure of myelinated neurons, which have an abundant number of sodium channels that enable membrane depolarization during an action potential, permitting the rapid saltatory propagation of electric signals across long axonal distance. Axonal degeneration is frequently observed in peroxisomal disorders such as adrenomyeloneuropathy (the late-onset variant of X-ALD). This peroxisomal dysfunction and accumulation of lipid metabolites in myelinating cells lead to unstable paranodal nodes and loss of axonal support, leading to an increase in oxidative damage and, consequently, axon degeneration (Citkowitz and Holtzman, 1973; Kassmann et al., 2011).

### 6.1 Peroxisomes in brain development

Peroxisomal biogenesis disorders (PBDs) are a spectrum of autosomal recessive metabolic disorders characterized by abnormal peroxisome assembly and impaired peroxisomal function. PBDs are subdivided into Zellweger Spectrum disorders and Rhizomelic Chondrodysplasia Punctata (RCDP) type (Argyriou et al., 2016).

The clinical symptoms that comprise Zellweger syndrome (OMIM #601539**)** are Zellweger syndrome (ZS), neonatal adrenoleukodystrophy, and infantile Refsum disease. Categorizing Zellweger is challenging due to the 13 PEX genes (PEX1, PEX2, PEX3, PEX5, PEX6, PEX10, PEX11b, PEX12, PEX13, PEX14, PEX16, PEX19, PEX26) that are known to be associated with the Peroxisome biogenesis disorders spectrum (Berger et al., 2016). A genotype-phenotype correspondence has been evaluated for PEX gene mutations, linking the nature and location of the mutation to the peroxin contribution to the metabolic functions of peroxisomes in patients (Steinberg et al., 2006). The neurological manifestation varies from primarily neurodevelopmental in the most severe to mainly degenerative in the milder cases (Barth et al., 2004). Zellweger syndrome (ZS) patients suffer from cortical malformations that have been linked primarily for defects in neuronal migration. These malformations have been observed in both hemispheres of the cerebral cortex, the cerebellum, and the inferior olivary complex. These migration defects lead to a local thickening of small convolutions (gyri) on the brain's surface that occurs around the central sulcus (centrosylvian pachygyria), causing a reduced depth of the fissions/involutions. Neurons destined to outer cortical layers II and III are the neuronal subtypes that are mainly affected in Zellweger syndrome patients. Interestingly, not all neurons are affected, as some neurons reach their regular laminar positions while others lie in the heterotopic intra-cortical or subcortical regions. One explanation for this incomplete disruption of neuronal migration could be linked to the presence of circulating toxic metabolites that accumulate due to altered peroxisomal functions (Evrard et al., 1978; Volpe and Adams, 1972). However, a recent metabolic study done on fibroblasts from patients that have dysfunctions in peroxisomes and mitochondria showed that manipulating a mitochondrial quality control factor ATAD1 was sufficient to restore mitochondrial dysfunction and metabolism in the fibroblasts (Nuebel et al., 2021). In Zellweger Syndrome, there is another striking morphological defect, where heterotopic Purkinje cells are localized in the white matter in the cerebellum; this defect is linked to abnormalities in neuronal migration (Evrard et al., 1978; Powers and Moser, 1998; Volpe and Adams, 1972). It is necessary to conduct a more extensive metabolic investigation of Zellweger patients' brains to better define the link between signaling molecules and neuronal migration (Jansen et al., 2000).

Mouse models to study the Zellweger spectrum are available, where mice carrying mutant Pex2, Pex13, and Pex5 resemble severe forms of human Zellweger syndrome. Mice born with these mutations survive up to 6–24 postnatally, and they show a severe form of growth retardation and hypotonia (Baes and Aubourg, 2009; Faust and Hatten, 1997; Maxwell et al., 2003). All the mice with global peroxisome deficiencies showed a reduction in cortical thickness that correlates with impairments in neuronal migration and an increase in heterotopias in white matter. Cerebellar deformities were only explored in the Pex2 mouse, as the cerebellum mainly develops postnatally (Faust et al., 2005).

Modifying the genetic background of Pex2 mice increases their survival rate by about 2 weeks. Moreover, postnatal survival and cerebellar deformities were improved in Pex2 pups by administration of oral bile acid. These findings point to the role of brain-extrinsic effects (effects originating from outside the brain) in brain development in peroxisomal disorders (Faust et al., 2005; Keane et al., 2007). There is a downside to bile oral treatment as it further perturbs the degree of hepatic steatosis and worsens the already severe mitochondrial and cellular damage in the peroxisome-deficient liver (Keane et al., 2007).

X-linked adrenoleukodystrophy results from mutations, deletions, missense, non-sense frameshifts, and splice defects involving the ABCD1 (adenosine triphosphate binding cassette) protein. This protein is absent in 70% of X-linked adrenoleukodystrophy patients (Farrell, 2012). ABCD1 gene defect leads to the accumulation of straight, saturated, very-long-chain fatty acids (those containing more than 22 carbons) in nervous and peripheral tissues and organs, eventually leading to one of the main phenotypes adrenomyeloneuropathy (AMN) or cerebral ALD (CALD), the severe inflammatory and demyelinating form of X-ALD. All male patients with mutations in the ABCD1 gene eventually develop adrenomyeloneuropathy, a slowly progressive myelopathy with a typical onset in the third or fourth decade of life (Singh, 1997). The earliest symptoms are usually urge incontinence and sensory disturbances in the legs, followed by spastic gait. The major neuropathological feature in adrenomyeloneuropathy is a distal dying-back axonopathy, which involves the dorsal columns and corticospinal tracts in the lower thoracic and lumbar regions (Dubey, 2005).

About 60% of male X-ALD patients develop CALD, the fatal cerebral demyelinating form of the disease. This can occur either in childhood, most commonly between 5 and 10 years of age, before onset of AMN (about 35%), or later in adolescence or adulthood, often on the background of AMN (35%). In children, the first symptoms are emotional lability, hyperactive behavior, school difficulties, impaired auditory discrimination, and vision difficulties. These early clinical symptoms are not specific, and the correct diagnosis of X-ALD is often delayed. This phase is followed by a rapidly progressing neurological decline, typically leading to a vegetative state or death within two to 5 years (Engelen et al., 2012). The disease development in X-ALD is associated with the cytotoxic effect due to the accumulation of very-long-chain fatty acids VLCFA (metabolic disease) and the subsequent triggering of an inflammatory cascade (inflammatory disease) that ultimately leads to demyelination and loss of oligodendrocytes. The observation of increased accumulation of very-long-chain fatty acids in inflammatory areas than in histologically normal areas of X-ALD brain (3-folds) suggests that inflammatory mediators downregulate peroxisomes/peroxisomal functions, thus creating a never-ending inflammatory cascade (Singh, 1997).

Interestingly, Abcd1 deficiency could enhance microglia activation and axonal degeneration in mice with mild myelin abnormalities caused by the loss of the myelin-associated glycoprotein (Mag) (Dumser et al., 2007). Intriguingly, Abcd1/Abcd2 Knockout mice do not develop brain inflammation or demyelination, despite the finding that Abcd1/Abcd2 double-deficient peritoneal macrophages are metabolically much more severely affected than those from single transporter deficient mice. Also, in these mice, the neuropathology is restricted mainly to axonopathy in the spinal cord and, with the major contribution from Abcd2 deficiency, the dorsal root ganglia, resulting in sensory neuropathy (Pujol et al., 2004). The current understanding is that the accumulation of VLCFA due to ABCD deficiency, especially in neurons and oligodendrocytes, is the main cause of the slow progression of chronic myeloneuropathy (AMN) (Manor et al., 2021).

The genetic cause for classical *Refsum disease* has been identified as mutations in gene encoding phytanoyl-CoA hydroxylase PHYH (Jansen et al., 2000). Patients with Refsum disease suffer from the accumulation of 3-methyl branched-chain fatty acid phytanic acid, which is solely taken up from dietary sources (Baldwin et al., 2010). Refsum disease is characterized by progressive retinitis pigmentosa culminating in blindness, peripheral neuropathy, and cerebellar ataxia. Mouse model of Refsum disease with mutation targeting Phyh gene did not show a significant phenotype due to the low branched-chain fatty acid in mouse food. However, additional supplementation with 0.25%–0.1% phytol (phytol precursor) for 3 weeks caused ataxia, reflecting Purkinje cell loss and astrogliosis in the cerebellum, and peripheral neuropathy, as revealed by nerve conduction velocity measurements (Ferdinandusse et al., 2008).

### 6.2 Peroxisomes in neurodegeneration

Peroxisome activity have been reported to play a role in neurodegenerative diseases, such as AD, where b amyloid and tau accumulate in neurons (Table 1).

A study showed that treating hippocampal neurons overexpressing β amyloid with a peroxisomal proliferator (Wy-14.463) increased the number of peroxisomes and catalase activity, reduced ROS production, and reduced overall degenerative symptoms such as calcium increase and beta-catenin instability (Santos et al., 2005).

Plasmalogens are among the most common glycerophospholipids; they are synthesized by peroxisomes and are among the major lipid components of these membranes. The highest amount of plasmalogen is found in the brain (Bozelli et al., 2021). Plasmalogens have been recently linked to degenerative and metabolic diseases in a clinical study on post-mortem samples where Alzheimer's patient's brains showed a shorter plasmalogens' half-life and a reduction in peroxisomal activity (Senanayake and Goodenowe, 2019). Data gathered so far suggest that the generalized impairment in peroxisome activity in AD might contribute to the disturbance of plasmalogen homeostasis. This hypothesis is supported by findings of altered levels of other peroxisomal metabolites in AD patients, such as increased VLCFA levels detected in cortical tissues and in the peripheral blood of AD patients (Wood et al., 2016). A recent study showed that the accumulation of peroxisomes in the soma of neurons in the gyrus frontalis of AD patients is paralleled by a lack of peroxisomes in dendrites with abnormally phosphorylated Tau protein, which might prevent the transport of peroxisomes into these processes. These results are complemented by studies in mouse models of AD showing that the number and protein content of peroxisomes correlate with disease progression, and changes in activity are mainly triggered by excessive oxidative stress and mitochondrial dysfunction (Kou et al., 2011).

In humans, peroxisomes contain D-amino acid oxidase (DAO), an enzyme involved in nutritional supply. These enzymes are flavin adenine dinucleotide (FAD)-containing flavoenzymes that oxidize certain amino acids, generating H2O2 as a side product. DAO activity is abundant in various human brain areas, but in the murine brain, the corresponding regions show markedly lower activity (Sasabe et al., 2014). This enzyme modulates the amount of neuroactive D-amino acids, D-serine, and D-aspartate (Oliet and Mothet, 2009). In the brain, D-serine is generated in astrocytes and released into the synaptic cleft but also taken up from the synaptic cleft and degraded in astrocytic peroxisomes. Peroxisome dysfunction has been reported in ALS through a genetic mutation in DAO (Kondori et al., 2017; 2018). Consistent with that, in a mouse model of familial ALS (SOD1G93A), the DAO activity in the spinal cord was reduced in parallel with an increase in D-serine levels.

There is a lot of evidence on the critical role of peroxisomes in the maintenance of CNS; they play a critical role in a variety of metabolic processes, especially in lipid metabolism, rendering them essential for human health and development. This makes peroxisomes a good candidate to investigate the treatment of neurological diseases.

## 7 Synaptic vesicles

**FIGURE 7:**
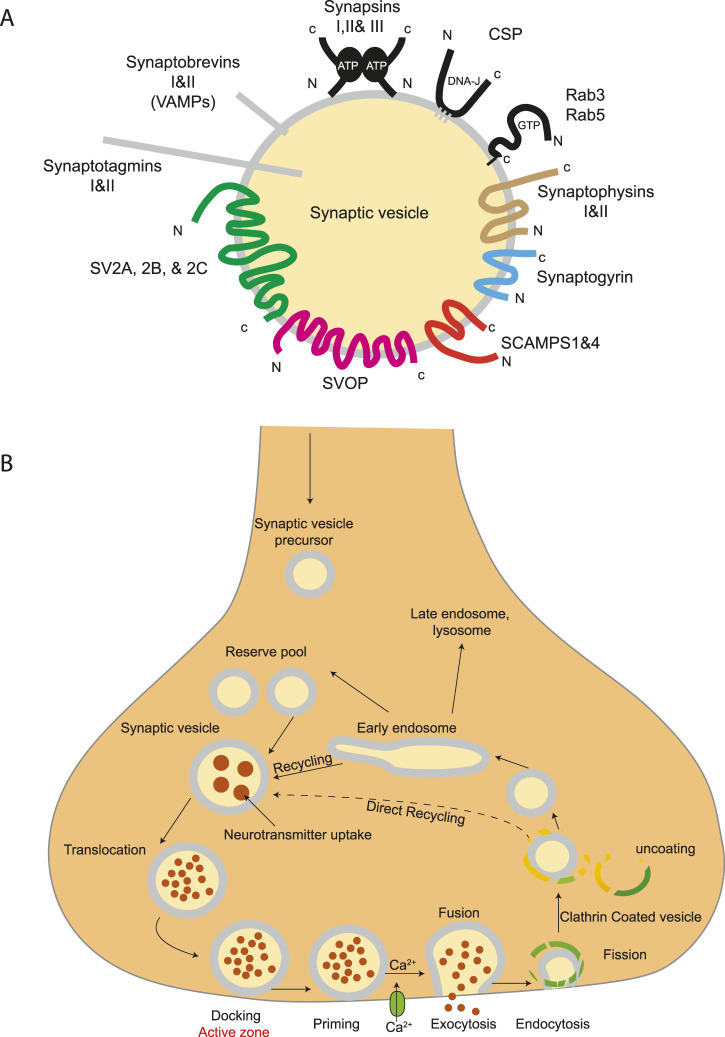
Structure of synaptic vesicles and trafficking pathway on nerve terminals. **(A)** A diagram showing the protein families belonging to the core set of synaptic vesicle proteins. Synaptic vesicles are small, membrane-enclosed organelles within nerve cells that store and release neurotransmitters. They are characterized by a uniform size, typically around 40 nm in diameter, and contain a limited number of specific proteins and lipids. Only proteins tightly associated with SVs are illustrated. **(B)** Trafficking pathways in the nerve terminal. Synaptic vesicles are filled with neurotransmitter and stored in the cytoplasm. Active vesicles are translocated to release sites in the active zone where they dock. Priming involves all steps required to acquire release readiness of the exocytotic complex. Although usually assumed to occur after docking, priming and even triggering may precede docking during sustained activity, resulting in immediate fusion of an arriving vesicle. After exocytosis, the vesicle proteins probably remain clustered and are then retrieved by endocytosis. Despite some lingering controversies, consensus is emerging that retrieval is generally mediated by clathrin-mediated endocytosis. After clathrin uncoating, synaptic vesicles are regenerated within the nerve terminal, probably involving passage through an endosomal intermediate. Actively recycling vesicles are in slow exchange with the reserve pool.

In neurons, neurotransmitter release is controlled by the release and recycling of the synaptic vesicles (SVs) (Figures 7A,B), a process tightly coordinated with neuronal activity, both temporally and spatially. The formation of a functional synapse undergoes three major steps: (1) neuronal contact formation triggering a cascade of cell adhesion molecules, (2) transport of synaptic components, with pre-and postsynaptic proteins synthesized and transported to sites of contact between axons and dendrites, and (3) stabilization of the synaptic components at the nascent synaptic contact sites (McAllister, 2007). In this section, we will discuss SV cycling, trafficking, and its implications for neurodevelopmental disorders (NDDs) and neurodegenerative disease.

### 7.1 SVs in brain development

Mutations in SVs cycling genes are considered among the causative genes in neurodevelopmental disorders (NDDs) (Washbourne, 2015). The correlation between NDDS, such as autism spectrum disorder (ASD) and intellectual disability (ID), was established as the appearance of NDDs symptoms occurs during the first 2 years of child development, corresponding to the period of massive synaptogenesis in the developing cortex (Huttenlocher and Dabholkar, 1997) (Table 1).

Kinesin-like proteins are axonal transporters of synaptic vesicles. They are microtubule-based proteins that belong to the kinesin protein family and are involved in the anterograde transport of proteins, organelles, and cargos of different nature (vesicles, macro-proteins, and mRNAs) along microtubules. In addition to its role in meiosis and mitosis, KIF1A gene plays a critical role in axonal transport. KIF1A is also required for neuronal dense core vesicles (DCV) transport to the dendritic spines and axons (Aguilera et al., 2021). Mutations in the KIF1A gene primarily affect the motor domain, disrupting synaptic vesicle transport containing synaptophysin and synaptotagmin. This disruption causes many neurological pathologies such as hereditary sensory neuropathy, autosomal dominant and recessive forms of spastic paraplegia, autism, neurodegeneration, and spasticity with or without cerebellar atrophy or cortical visual impairment (NESCAV syndrome) (Nair et al., 2023). KIF1A neurological disorder (KAND) patients are identified in early childhood and have a life expectancy of 5–7 years. Functional studies performed on hippocampal neurons transfected with *de novo* missense variants (T99M, A202P, S215, R216P, and E253K) in the motor domain of Kif1a showed a reduced distal localization in neurites of the mutant protein compared to the wildtype (Lee J. R. et al., 2015). Furthermore, experiments performed in SH-SY5Y cells demonstrated that the *de novo* missense variants of KIF1A led to an accumulation of mutated protein preferentially in the cell body instead of the tips of neurites, where wild-type KIF1A is typically located (Kaur et al., 2020).

Microtubule-associated protein tau (MAPT) is a gene responsible for encoding tau protein, tightly implicated in keeping the function of microtubules and axonal transport, as microtubules act as the rails where long-distance axonal transport occurs. MAPT promotes microtubule assembly and stability and may act as a linker protein between the microtubules and the plasma membrane. Mutations affecting MAPT gene on chromosome 17 have been associated with NDDs, mainly with intellectual disabilities and developmental delay (Washbourne, 2015). Results obtained from developing mouse brain upon Tau reduction show an impaired neuronal migration and less developed dendrites with an aberrant reduction in connectivity for the neurons reaching the cortical plate. Furthermore, intracellular mitochondrial transport and morphology were disrupted (Sapir et al., 2012).

### 7.2 SVs in neurodegeneration

Microtubule-associated protein tau has been commonly associated with neurodegenerative diseases: hyperphosphorylated tau proteins are involved in the formation of neurofibrillary tangles (NFTs), which characterize many neurodegenerative disorders classified as tauopathies. Genome-wide association studies (GWAS) identified several single nucleotide polymorphisms (SNPs) that are in the MAPT locus and are associated with various neurodegenerative diseases (Ma et al., 2018). A possible mechanism leading to neurodegeneration is suggested by the observation that overexpression of tau and mislocalization to the somatodendritic compartment impair the axonal transport of mitochondria. One can speculate that cell death is directly linked to the shortage of general energy supply to the neuron and to the axon and synaptic boutons, subcellular compartments with very high energy demand (Tracy et al., 2022).

Vesicular trafficking and fusion events are modulated by GDP-dissociation inhibitor 1 (GDI1) *via* the regulation of Rab GTPase activity (Bächner et al., 1995). GDI1 is expressed mainly in neural and sensory tissues, and pathogenic variants of GDI1 have been associated with Mental retardation and pronounced speech delay (Bienvenu et al., 1998). GDI1-deficient mice suffer from defects in short-term temporal associations, suggesting a defect in short-term memory. This is reminiscent of the situation in human patients, where a lack of GDI1 impairs memory formation in the forebrain (D'Adamo et al., 2002). High levels of GDI1 were observed in the serum of AD patients and Aβ-induced SH-SY5Y cells. Attenuating the level of GDI1 in Aβ-induced SH-SY5Y cells decreased inflammation, apoptosis, and neurotoxicity. These results put GDI1 as a potential biomarker for AD (Liu et al., 2024). The increased understanding of basic cell biological mechanisms underlying NDDs and neurodegenerative clinical phenotypes may translate into personalized clinical management and improved quality of life for patients and families.

## 8 Concluding remarks

The study of vesicle trafficking and its related disorders presents a major challenge but also offers an opportunity to unlock new frontiers by bridging cell biology, biochemistry, metabolism, and genetic medicine. Given the wide variety of diseases and the complexity of the biological processes involved, many key questions remain unanswered. How are transport intermediates, such as synaptic vesicles, secretory vesicles, lysosomes, extracellular vesicles, and peroxisomes, organized at various cellular interfaces and in different cell types? Since most mutations associated with these diseases result in a loss of function, how will this impact and inform potential therapeutic strategies? The pivotal role of vesicle trafficking in the nervous system, alongside the unique vulnerability of neurons to trafficking defects, raises important questions. This is particularly true for neurodegenerative and neurodevelopmental diseases, where disruptions in vesicle trafficking affecting structures like synaptic vesicles or lysosomes have profound effects on brain development and function.

In this context, the identification of biomarkers to detect dysfunctions in these pathways could lead to earlier diagnosis and personalized treatments. Furthermore, patient-derived induced pluripotent stem cells (iPSCs) and organoid models offer valuable platforms for studying these mechanisms in a more physiologically relevant environment, providing powerful tools to deepen our understanding of these complex disorders. The increased understanding of basic cell biological mechanisms underlying neurodevelopmental disorders and neurodegenerative clinical phenotypes may translate into personalized clinical management and improved quality of life for patients and families.

## References

[B1] Abela L. Gianfrancesco L. Tagliatti E. Rossignoli G. Barwick K. Zourray C. (2024). Neurodevelopmental and synaptic defects in DNAJC6 parkinsonism, amenable to gene therapy. Brain 147 (6), 2023–2037. 10.1093/brain/awae020 38242634 PMC11146427

[B2] Aguilera C. Hümmer S. Masanas M. Gabau E. Guitart M. Jeyaprakash A. A. (2021). The novel KIF1A missense variant (R169T) strongly reduces microtubule stimulated ATPase activity and is associated with NESCAV syndrome. Front. Neurosci. 15, 618098–618099. 10.3389/fnins.2021.618098 34121983 PMC8187576

[B3] Arakel E. C. Schwappach B. (2018). Correction: formation of COPI-coated vesicles at a glance. J. Cell Sci. 131 (7), jcs209890. 10.1242/jcs.209890 29632048

[B4] Argyriou C. D'Agostino M. D. Braverman N. (2016). Peroxisome biogenesis disorders. Transl. Sci. Rare Dis. 1 (2), 111–144. 10.3233/trd-160003 29152457 PMC5678237

[B5] Arnold G. Liscum L. Holtzman E. (1979). Ultrastructural localization of D-amino acid oxidase in microperoxisomes of the rat nervous system. J. Histochem. Cytochem. 27 (3), 735–745. 10.1177/27.3.39097 39097

[B6] Bächner D. Sedlacek Z. Korn B. Hameister H. Poustka A. (1995). Expression patterns of two human genes coding for different rab GDP-dissociation inhibitors (GDIs), extremely conserved proteins involved in cellular transport. Hum. Mol. Genet. 4 (4), 701–708. 10.1093/hmg/4.4.701 7543319

[B7] Baes M. Aubourg P. (2009). Peroxisomes, myelination, and axonal integrity in the CNS. Neuroscientist 15 (4), 367–379. 10.1177/1073858409336297 19666893

[B8] Baldwin E. J. Gibberd F. B. Harley C. Sidey M. C. Feher M. D. Wierzbicki A. S. (2010). The effectiveness of long-term dietary therapy in the treatment of adult Refsum disease. J. Neurology, Neurosurg. Psychiatry 81 (9), 954–957. 10.1136/jnnp.2008.161059 20547622

[B9] Ballabio A. Bonifacino J. S. (2020). Lysosomes as dynamic regulators of cell and organismal homeostasis. Nat. Rev. Mol. Cell Biol. 21 (2), 101–118. 10.1038/s41580-019-0185-4 31768005

[B10] Barth P. G. Majoie C. B. L. M. Gootjes J. Wanders R. J. A. Waterham H. R. van der Knaap M. S. (2004). Neuroimaging of peroxisome biogenesis disorders (*Zellweger spectrum*) with prolonged survival. Neurology 62 (3), 439–444. 10.1212/01.WNL.0000106943.40848.03 14872027

[B11] Bazinet R. P. Layé S. (2014). Polyunsaturated fatty acids and their metabolites in brain function and disease. Nat. Rev. Neurosci. 15 (12), 771–785. 10.1038/nrn3820 25387473

[B12] Bentley M. Banker G. (2016). The cellular mechanisms that maintain neuronal polarity. Nat. Rev. Neurosci. 17 (10), 611–622. 10.1038/nrn.2016.100 27511065

[B13] Berger J. Dorninger F. Forss-Petter S. Kunze M. (2016). Peroxisomes in brain development and function. Biochimica Biophysica Acta - Mol. Cell Res. 1863 (5), 934–955. 10.1016/j.bbamcr.2015.12.005 PMC4880039 26686055

[B14] Bettayeb K. Chang J. C. Luo W. Aryal S. Varotsis D. Randolph L. (2016). δ-COP modulates Aβ peptide formation via retrograde trafficking of APP. Proc. Natl. Acad. Sci. U. S. A. 113 (19), 5412–5417. 10.1073/pnas.1604156113 27114525 PMC4868462

[B15] Bienvenu T. Des Portes V. Saint Martin A. McDonell N. Billuart P. Carrié A. (1998). Non-specific X-linked semidominant mental retardation by mutations in a Rab GDP-dissociation inhibitor. Hum. Mol. Genet. 7 (8), 1311–1315. 10.1093/hmg/7.8.1311 9668174

[B16] Boyle B. R. Melli S. E. Altreche R. S. Padron Z. M. Yousufzai F. A. K. Kim S. (2020). NPC1 deficiency impairs cerebellar postnatal development of microglia and climbing fiber refinement in a mouse model of Niemann-Pick disease type C. Dev. Camb. 147 (21), dev189019–13. 10.1242/dev.189019 PMC7420841 32611604

[B17] Bozelli J. C. Azher S. Epand R. M. (2021). Plasmalogens and chronic inflammatory diseases. Front. Physiology 12 (October), 730829–730919. 10.3389/fphys.2021.730829 PMC8566352 34744771

[B18] Brault J. Bardin S. Lampic M. Carpentieri J. A. Coquand L. Penisson M. (2022). RAB6 and dynein drive post‐Golgi apical transport to prevent neuronal progenitor delamination. EMBO Rep. 23 (10), e54605. 10.15252/embr.202254605 35979738 PMC9535803

[B19] Braverman N. E. D'Agostino M. D. MacLean G. E. (2013). Peroxisome biogenesis disorders: biological, clinical and pathophysiological perspectives. Dev. Disabil. Res. Rev., 17(3), 187–196. 10.1002/ddrr.1113 23798008

[B20] Britt D. J. Farías G. G. Guardia C. M. Bonifacino J. S. (2016). Mechanisms of polarized organelle distribution in neurons. Front. Cell. Neurosci. 10 (MAR2016), 88–97. 10.3389/fncel.2016.00088 27065809 PMC4814528

[B21] Buratta S. Tancini B. Sagini K. Delo F. Chiaradia E. Urbanelli L. (2020). Lysosomal exocytosis, exosome release and secretory autophagy: the autophagic‐ and endo-lysosomal systems go extracellular. Int. J. Mol. Sci. 21 (2576), 1–20. 10.3390/ijms21072576 PMC7178086 32276321

[B22] Cai Z. Y. Xiao M. Quazi S. Ke Z. Y. (2018). Exosomes: a novel therapeutic target for Alzheimer's disease? Neural Regen. Res. 13 (5), 930–935. 10.4103/1673-5374.232490 29863025 PMC5998631

[B23] Caporali P. Bruno F. Palladino G. Dragotto J. Petrosini L. Mangia F. (2016). Developmental delay in motor skill acquisition in niemann-pick C1 mice reveals abnormal cerebellar morphogenesis. Acta Neuropathol. Commun. 4 (1), 94–18. 10.1186/s40478-016-0370-z 27586038 PMC5009663

[B24] Choy R. W. Y. Cheng Z. Schekman R. (2012). Amyloid precursor protein (APP) traffics from the cell surface via endosomes for amyloid β (Aβ) production in the trans-Golgi network. Proc. Natl. Acad. Sci. U. S. A. 109 (30), 2077–2082. 10.1073/pnas.1208635109 PMC3409748 22711829

[B25] Citkowitz E. Holtzman E. (1973). Peroxisomes in dorsal root ganglia. J. Histochem. Cytochem. 21 (1), 34–41. 10.1177/21.1.34 4694538

[B26] Coen K. Flannagan R. S. Baron S. Carraro-Lacroix L. R. Wang D. Vermeire W. (2012). Lysosomal calcium homeostasis defects, not proton pump defects, cause endo-lysosomal dysfunction in PSEN-deficient cells. J. Cell Biol. 198 (1), 23–35. 10.1083/jcb.201201076 22753898 PMC3392942

[B27] Cooper G. M. Adams K. (2022). The cell: a molecular approach 7th ed. Sunderland, MA: Sinauer.

[B28] Credle J. J. Forcelli P. A. Delannoy M. Oaks A. W. Permaul E. Berry D. L. (2015). α-Synuclein-mediated inhibition of ATF6 processing into COPII vesicles disrupts UPR signaling in Parkinson's disease. Neurobiol. Dis. 76, 112–125. 10.1016/j.nbd.2015.02.005 25725420

[B29] D'Adamo P. Welzl H. Papadimitriou S. Raffaele di Barletta M. Tiveron C. Tatangelo L. (2002). Deletion of the mental retardation gene Gdi1 impairs associative memory and alters social behavior in mice. Hum. Mol. Genet. 11 (21), 2567–2580. 10.1093/hmg/11.21.2567 12354782

[B30] Danzer K. M. Kranich L. R. Ruf W. P. Cagsal-Getkin O. Winslow A. R. Zhu L. (2012). Exosomal cell-to-cell transmission of alpha synuclein oligomers. Mol. Neurodegener. 7 (1), 42–18. 10.1186/1750-1326-7-42 22920859 PMC3483256

[B31] de Almeida Fuzeta M. Bernardes N. Oliveira F. D. Costa A. C. Fernandes-Platzgummer A. Farinha J. P. (2020). Scalable production of human mesenchymal stromal cell-derived extracellular vesicles under serum-/xeno-free conditions in a microcarrier-based bioreactor culture system. Front. Cell Dev. Biol. 8 (November), 553444. 10.3389/fcell.2020.553444 33224943 PMC7669752

[B32] Dell'Angelica E. C. Bonifacino J. S. (2019). Coatopathies: genetic disorders of protein coats. Annu. Rev. Cell Dev. Biol. 35, 131–168. 10.1146/annurev-cellbio-100818-125234 31399000 PMC7310445

[B33] De Toro J. Herschlik L. Waldner C. Mongini C. (2015). Emerging roles of exosomes in normal and pathological conditions: new insights for diagnosis and therapeutic applications. Front. Immunol. 6 (MAY), 203–212. 10.3389/fimmu.2015.00203 25999947 PMC4418172

[B34] DiStasio A. Driver A. Sund K. Donlin M. Muraleedharan R. M. Pooya S. (2017). Copb2 is essential for embryogenesis and hypomorphic mutations cause human microcephaly. Hum. Mol. Genet. 26 (24), 4836–4848. 10.1093/hmg/ddx362 29036432 PMC5886270

[B35] Dubey P. Raymond G. V. Moser A. B. Kharkar S. Bezman L. Moser H. W. (2005). Adrenal insufficiency in asymptomatic adrenoleukodystrophy patients identified by very long-chain fatty acid screening. J. Pediatr. 146 (4), 528–532. 10.1016/j.jpeds.2004.10.067 15812458

[B36] Dubreuil V. Marzesco A. M. Corbeil D. Huttner W. B. Wilsch-Bräuninger M. (2007). Midbody and primary cilium of neural progenitors release extracellular membrane particles enriched in the stem cell marker prominin-1. J. Cell Biol. 176 (4), 483–495. 10.1083/jcb.200608137 17283184 PMC2063983

[B37] Dumser M. Bauer J. Lassmann H. Berger J. Forss-Petter S. (2007). Lack of adrenoleukodystrophy protein enhances oligodendrocyte disturbance and microglia activation in mice with combined Abcd1 Mag deficiency. Acta Neuropathol. 114 (6), 573–586. 10.1007/s00401-007-0288-4 17828604

[B38] Emmanouilidou E. Melachroinou K. Roumeliotis T. Garbis S. D. Ntzouni M. Margaritis L. H. (2010). Cell-produced α-synuclein is secreted in a calcium-dependent manner by exosomes and impacts neuronal survival. J. Neurosci. 30 (20), 6838–6851. 10.1523/JNEUROSCI.5699-09.2010 20484626 PMC3842464

[B39] Engelen M. Kemp S. De Visser M. Van Geel B. M. Wanders R. J. A. Aubourg P. (2012). X-linked adrenoleukodystrophy (X-ALD): clinical presentation and guidelines for diagnosis, follow-up and management. Orphanet J. Rare Dis. 7 (1), 51–14. 10.1186/1750-1172-7-51 22889154 PMC3503704

[B40] Ettinger A. W. Wilsch-Bräuninger M. Marzesco A. M. Bickle M. Lohmann A. Maliga Z. (2011). Proliferating versus differentiating stem and cancer cells exhibit distinct midbody-release behaviour. Nat. Commun. 2 (1), 503. 10.1038/ncomms1511 22009035 PMC3207209

[B41] Evrard P. Caviness V. S. Prats-Vinas J. Lyon G. (1978). The mechanism of arrest of neuronal migration in the Zellweger malformation: an hypothesis bases upon cytoarchitectonic analysis. Acta Neuropathol. 41 (2), 109–117. 10.1007/BF00689761 636841

[B42] Farrell D. F. (2012). Neonatal adrenoleukodystrophy: a clinical, pathologic, and biochemical study. Pediatr. Neurol. 47 (5), 330–336. 10.1016/j.pediatrneurol.2012.07.006 23044013

[B43] Fauré J. Lachenal G. Court M. Hirrlinger J. Chatellard-Causse C. Blot B. (2006). Exosomes are released by cultured cortical neurones. Mol. Cell. Neurosci. 31 (4), 642–648. 10.1016/j.mcn.2005.12.003 16446100

[B44] Faust P. L. Banka D. Siriratsivawong R. Ng V. G. Wikander T. M. (2005). Peroxisome biogenesis disorders: the role of peroxisomes and metabolic dysfunction in developing brain. J. Inherit. Metabolic Dis. 28 (3), 369–383. 10.1007/s10545-005-7059-y 15868469

[B45] Faust P. L. Hatten M. E. (1997). Targeted deletion of the PEX2 peroxisome assembly gene in mice provides a model for Zellweger syndrome, a human neuronal migration disorder. J. Cell Biol. 139 (5), 1293–1305. 10.1083/jcb.139.5.1293 9382874 PMC2140200

[B46] Ferdinandusse S. Zomer A. W. M. Komen J. C. Van Den Brink C. E. Thanos M. Hamers F. P. T. (2008). Ataxia with loss of Purkinje cells in a mouse model for Refsum disease. Proc. Natl. Acad. Sci. U. S. A. 105 (46), 17712–17717. 10.1073/pnas.0806066105 19004801 PMC2584743

[B47] Fernández V. Llinares‐Benadero C. Borrell V. (2016). Cerebral cortex expansion and folding: what have we learned? EMBO J. 35 (10), 1021–1044. 10.15252/embj.201593701 27056680 PMC4868950

[B48] Fiandaca M. S. Kapogiannis D. Mapstone M. Boxer A. Eitan E. Schwartz J. B. (2015). Identification of preclinical Alzheimer's disease by a profile of pathogenic proteins in neurally derived blood exosomes: a case-control study. Alzheimer's Dementia J. Alzheimer's Assoc. 11 (6), 600–607.e1. 10.1016/j.jalz.2014.06.008 PMC4329112 25130657

[B49] Filaquier A. Marin P. Parmentier M. L. Villeneuve J. (2022). Roads and hubs of unconventional protein secretion. Curr. Opin. Cell Biol. 75, 102072. 10.1016/j.ceb.2022.02.006 35305454

[B50] Florio M. Huttner W. B. (2014). Neural progenitors, neurogenesis and the evolution of the neocortex. Dev. Camb. 141 (11), 2182–2194. 10.1242/dev.090571 24866113

[B51] Fontaine S. N. Zheng D. Sabbagh J. J. Martin M. D. Chaput D. Darling A. (2016). DnaJ/Hsc70 chaperone complexes control the extracellular release of neurodegenerative‐associated proteins. EMBO J. 35 (14), 1537–1549. 10.15252/embj.201593489 27261198 PMC4946142

[B52] Forero A. Pipicelli F. Moser S. Baumann N. Grätz C. Gonzalez Pisfil M. (2024). Extracellular vesicle-mediated trafficking of molecular cues during human brain development Extracellular vesicle-mediated trafficking of molecular cues during human brain development. Cell Rep. 43 (10), 114755. 10.1016/j.celrep.2024.114755 39302835

[B53] Ganesan H. Balasubramanian V. Iyer M. Venugopal A. Subramaniam M. D. Cho S. G. (2019). mTOR signalling pathway - a root cause for idiopathic autism? BMB Rep. 52 (7), 424–433. 10.5483/BMBRep.2019.52.7.137 31186084 PMC6675248

[B54] Garcia-Contreras M. Thakor A. S. (2023). Extracellular vesicles in Alzheimer's disease: from pathology to therapeutic approaches. Neural Regen. Res. 18 (1), 18–22. 10.4103/1673-5374.343882 35799503 PMC9241420

[B55] Geffen Y. Perets N. Horev R. Yudin D. Oron O. Elliott E. (2020). Exosomes derived from adipose mesenchymal stem cells: a potential non-invasive intranasal treatment for autism. Cytotherapy 22 (5), S49. 10.1016/j.jcyt.2020.03.059

[B56] Giraudo C. G. Daniotti J. L. Maccioni H. J. F. (2001). Physical and functional association of glycolipid N-acetyl-galactosaminyl and galactosyl transferases in the Golgi apparatus. Proc. Natl. Acad. Sci. U. S. A. 98 (4), 1625–1630. 10.1073/pnas.98.4.1625 11172001 PMC29307

[B57] Gomes A. R. Sangani N. B. Fernandes T. G. Diogo M. M. Curfs L. M. G. Reutelingsperger C. P. (2020). Extracellular vesicles in cns developmental disorders. Int. J. Mol. Sci. 21 (24), 9428–9520. 10.3390/ijms21249428 33322331 PMC7763819

[B58] Gonatas N. K. Stieber A. Mourelatos Z. Chen Y. Gonatas J. O. Appel S. H. (1992). Fragmentation of the Golgi apparatus of motor neurons in amyotrophic lateral sclerosis. Am. J. Pathol. 140 (3), 731–737. 1546747 PMC1886164

[B59] Haass C. Kaether C. Thinakaran G. Sisodia S. (2012). Trafficking and proteolytic processing of APP. Cold Spring Harb. Perspect. Med. 2 (5), 0062700–a6325. 10.1101/cshperspect.a006270 PMC3331683 22553493

[B60] Hamlett D. Boger H. Ledreux A. M. Kelley C. J. Mufson E. F. Falangola M. N. (2015). Cognitive impairment, neuroimaging, and alzheimer neuropathology in mouse models of Down syndrome. Curr. Alzheimer Res. 13 (1), 35–52. 10.2174/1567205012666150921095505 PMC5034871 26391050

[B61] Hamlett E. D. Goetzl E. J. Ledreux A. Vasilevko V. Boger H. A. LaRosa A. (2017). Neuronal exosomes reveal Alzheimer's disease biomarkers in Down syndrome. Alzheimer's Dementia 13 (5), 541–549. 10.1016/j.jalz.2016.08.012 PMC5812672 27755974

[B62] Hamlett E. D. Ledreux A. Potter H. Chial H. J. Patterson D. Espinosa J. M. (2018). Exosomal biomarkers in Down syndrome and Alzheimer's disease. Free Radic. Biol. Med. 114 (July 2017), 110–121. 10.1016/j.freeradbiomed.2017.08.028 28882786 PMC6135098

[B63] Hiltunen J. K. Mursula A. M. Rottensteiner H. Wierenga R. K. Kastaniotis A. J. Gurvitz A. (2003). The biochemistry of peroxisomal β-oxidation in the yeast *Saccharomyces cerevisiae* . FEMS Microbiol. Rev. 27 (1), 35–64. 10.1016/S0168-6445(03)00017-2 12697341

[B64] Holtzman D. M. Santucci D. Kilbridge J. Chua-Couzens J. Fontana D. J. Daniels S. E. (1996). Developmental abnormalities and age-related neurodegeneration in a mouse model of Down syndrome. Proc. Natl. Acad. Sci. U. S. A. 93 (23), 13333–13338. 10.1073/pnas.93.23.13333 8917591 PMC24093

[B65] Huttenlocher P. R. Dabholkar A. S. (1997). Regional differences in synaptogenesis in human cerebral cortex. J. Comp. Neurol. 387 (2), 167–178. 10.1002/(SICI)1096-9861(19971020)387:2<167::AID-CNE1>3.0.CO;2-Z 9336221

[B66] Ibáñez F. Montesinos J. Ureña-Peralta J. R. Guerri C. Pascual M. (2019). TLR4 participates in the transmission of ethanol-induced neuroinflammation via astrocyte-derived extracellular vesicles. J. Neuroinflammation 16 (1), 1–14. 10.1186/s12974-019-1529-x 31272469 PMC6610989

[B67] Iguchi Y. Eid L. Parent M. Soucy G. Bareil C. Riku Y. (2016). Exosome secretion is a key pathway for clearance of pathological TDP-43. Brain 139 (12), 3187–3201. 10.1093/brain/aww237 27679482 PMC5840881

[B68] Ishikawa T. Okada T. Ishikawa-Fujiwara T. Todo T. Kamei Y. Shigenobu S. (2013). ATF6α/β-mediated adjustment of ER chaperone levels is essential for development of the notochord in medaka fish. Mol. Biol. Cell 24 (9), 1387–1395. 10.1091/mbc.E12-11-0830 23447699 PMC3639050

[B69] Islinger M. Voelkl A. Fahimi H. D. Schrader M. (2018). The peroxisome: an update on mysteries 2.0. Histochem. Cell Biol. 150 (5), 443–471. 10.1007/s00418-018-1722-5 30219925 PMC6182659

[B70] Izumi K. Brett M. Nishi E. Drunat S. Tan E. S. Fujiki K. (2016). ARCN1 mutations cause a recognizable craniofacial syndrome due to COPI-mediated transport defects. Am. J. Hum. Genet. 99 (2), 451–459. 10.1016/j.ajhg.2016.06.011 27476655 PMC4974084

[B71] Jacopo M. (2023). Unconventional protein secretion (UPS): role in important diseases. Mol. Biomed. 4 (1), 2. 10.1186/s43556-022-00113-z 36622461 PMC9827022

[B72] Jansen G. A. Hogenhout E. M. Ferdinandusse S. Waterham H. R. Ofman R. Jakobs C. (2000). Human phytanoyl-CoA hydroxylase: resolution of the gene structure and the molecular basis of Refsum's disease. Hum. Mol. Genet. 9 (8), 1195–1200. 10.1093/hmg/9.8.1195 10767344

[B73] Jiang Y. Sato Y. Im E. Berg M. Bordi M. Darji S. (2019). Lysosomal dysfunction in down syndrome is app-dependent and mediated by APP-βCTF (c99). J. Neurosci. 39 (27), 5255–5268. 10.1523/JNEUROSCI.0578-19.2019 31043483 PMC6607756

[B74] Kalluri R. LeBleu V. S. (2020). The biology, function, and biomedical applications of exosomes. Sci. (New York, N.Y.) 367 (6478), eaau6977. 10.1126/science.aau6977 PMC7717626 32029601

[B75] Kassmann C. M. Quintes S. Rietdorf J. Möbius W. Sereda M. W. Nientiedt T. (2011). A role for myelin-associated peroxisomes in maintaining paranodal loops and axonal integrity. FEBS Lett. 585 (14), 2205–2211. 10.1016/j.febslet.2011.05.032 21620837

[B76] Kaur S. Van Bergen N. J. Verhey K. J. Nowell C. J. Budaitis B. Yue Y. (2020). Expansion of the phenotypic spectrum of *de novo* missense variants in kinesin family member 1A (KIF1A). Hum. Mutat. 41 (10), 1761–1774. 10.1002/humu.24079 32652677 PMC7908811

[B77] Keane M. H. Overmars H. Wikander T. M. Ferdinandusse S. Duran M. Wanders R. J. A. (2007). Bile acid treatment alters hepatic disease and bile acid transport in peroxisome-deficient PEX2 Zellweger mice. Hepatol. Baltim. Md 45 (4), 982–997. 10.1002/hep.21532 17393522

[B78] Kim S. Ochoa K. Melli S. E. Yousufzai F. A. K. Barrera Z. D. Williams A. A. (2023). Disruptive lysosomal-metabolic signaling and neurodevelopmental deficits that precede Purkinje cell loss in a mouse model of Niemann-Pick Type-C disease. Sci. Rep. 13 (1), 5665–5717. 10.1038/s41598-023-32971-0 37024714 PMC10079843

[B79] Kingma S. D. K. Bodamer O. A. Wijburg F. A. (2015). Epidemiology and diagnosis of lysosomal storage disorders; challenges of screening. Best Pract. Res. Clin. Endocrinol. Metabolism 29 (2), 145–157. 10.1016/j.beem.2014.08.004 25987169

[B80] Kittler R. Pelletier L. Heninger A. Slabicki M. Theis M. Miroslaw L. (2007). Genome-scale RNAi profiling of cell division in human tissue culture cells. Nat. Cell Biol. 9 (12), 1401–1412. 10.1038/ncb1659 17994010

[B81] Kondori N. R. Paul P. Robbins J. P. Liu K. Hildyard J. C. W. Wells D. J. (2017). Characterisation of the pathogenic effects of the *in vivo* expression of an ALS-linked mutation in D-amino acid oxidase: phenotype and loss of spinal cord motor neurons. PLoS One 12 (12), e0188912–e0188922. 10.1371/journal.pone.0188912 29194436 PMC5711026

[B82] Kondori N. R. Paul P. Robbins J. P. Liu K. Hildyard J. C. W. Wells D. J. (2018). Focus on the role of D-serine and D-amino acid oxidase in Amyotrophic Lateral Sclerosis/motor neuron disease (ALS). Front. Mol. Biosci. 5 (FEB), 8. 10.3389/fmolb.2018.00008 29487852 PMC5816792

[B83] Kou J. Kovacs G. G. Höftberger R. Kulik W. Brodde A. Forss-Petter S. (2011). Peroxisomal alterations in Alzheimer's disease. Acta Neuropathol. 122 (3), 271–283. 10.1007/s00401-011-0836-9 21594711 PMC3168371

[B84] Lachenal G. Pernet-Gallay K. Chivet M. Hemming F. J. Belly A. Bodon G. (2011). Release of exosomes from differentiated neurons and its regulation by synaptic glutamatergic activity. Mol. Cell. Neurosci. 46 (2), 409–418. 10.1016/j.mcn.2010.11.004 21111824

[B85] Laguesse S. Creppe C. Nedialkova D. D. Prévot P. P. Borgs L. Huysseune S. (2015). A dynamic unfolded protein response contributes to the control of cortical neurogenesis. Dev. Cell 35 (5), 553–567. 10.1016/j.devcel.2015.11.005 26651292

[B86] Lee J. H. McBrayer M. K. Wolfe D. M. Haslett L. J. Kumar A. Sato Y. (2015). Presenilin 1 maintains lysosomal Ca^2+^ homeostasis via TRPML1 by regulating vATPase-mediated lysosome acidification. Cell Rep. 12 (9), 1430–1444. 10.1016/j.celrep.2015.07.050 26299959 PMC4558203

[B87] Lee J. R. Srour M. Kim D. Hamdan F. F. Lim S. H. Brunel-Guitton C. (2015). *De novo* mutations in the motor domain of KIF1A cause cognitive impairment, spastic paraparesis, axonal neuropathy, and cerebellar atrophy. Hum. Mutat. 36 (1), 69–78. 10.1002/humu.22709 25265257

[B88] Liu L. Liu L. Lu Y. Zhang T. Zhao W. (2024). GDP dissociation inhibitor 1 (GDI1) attenuates β-amyloid-induced neurotoxicity in Alzheimer's diseases. Neurosci. Lett. 818 (766), 137564. 10.1016/j.neulet.2023.137564 38013121

[B89] Lodhi I. J. Semenkovich C. F. (2014). Peroxisomes: a nexus for lipid metabolism and cellular signaling. Cell Metab. 19 (3), 380–392. 10.1016/j.cmet.2014.01.002 24508507 PMC3951609

[B90] Lopez A. Siddiqi F. H. Villeneuve J. Ureshino R. P. Jeon H.-Y. Koulousakis P. (2024). Carbonic anhydrase inhibition ameliorates tau toxicity via enhanced tau secretion. Nat. Chem. Biol. 21, 577, 587. 10.1038/s41589-024-01762-7 39482469 PMC11949835

[B91] Lowe M. (2011). Structural organization of the Golgi apparatus. Curr. Opin. Cell Biol. 23 (1), 85–93. 10.1016/j.ceb.2010.10.004 21071196

[B92] Ma F. C. Wang H. F. Cao X. P. Tan C. C. Tan L. Yu J. T. (2018). Meta-Analysis of the association between variants in ABCA7 and Alzheimer's disease. J. Alzheimer's Dis. 63 (4), 1261–1267. 10.3233/JAD-180107 29782324

[B93] Macken W. L. Godwin A. Wheway G. Stals K. Nazlamova L. Ellard S. (2021). Biallelic variants in COPB1 cause a novel, severe intellectual disability syndrome with cataracts and variable microcephaly. Genome Med. 13 (1), 34–19. 10.1186/s13073-021-00850-w 33632302 PMC7908744

[B94] Mahnke A. H. Adams A. M. Wang A. Z. Miranda R. C. (2019). Toxicant and teratogenic effects of prenatal alcohol. Curr. Opin. Toxicol. 14, 29–34. 10.1016/j.cotox.2019.08.002 32864517 PMC7451635

[B95] Malhotra V. (2013). Unconventional protein secretion: an evolving mechanism. EMBO J. 32 (12), 1660–1664. 10.1038/emboj.2013.104 23665917 PMC3680731

[B96] Malhotra V. (2025). The pathways of secretory cargo export at the endoplasmic reticulum. Nat. Commun. 16 (1), 2138–2145. 10.1038/s41467-025-57408-2 40032897 PMC11876584

[B97] Manor J. Chung H. Bhagwat P. K. Wangler M. F. (2021). ABCD1 and X-linked adrenoleukodystrophy: a disease with a markedly variable phenotype showing conserved neurobiology in animal models. J. Neurosci. Res. 99 (12), 3170–3181. 10.1002/jnr.24953 34716609 PMC9665428

[B98] Marchetto M. C. N. Carromeu C. Acab A. Yu D. Yeo G. W. Mu Y. (2010). A model for neural development and treatment of rett syndrome using human induced pluripotent stem cells. Cell 143 (4), 527–539. 10.1016/j.cell.2010.10.016 21074045 PMC3003590

[B99] Martínez-Menárguez J. Á. Tomás M. Martínez-Martínez N. Martínez-Alonso E. (2019). Golgi fragmentation in neurodegenerative diseases: is there a common cause? Cells 8 (7), 1–19. 10.3390/cells8070748 PMC6679019 31331075

[B100] Marzesco A. M. Janich P. Wilsch-Bräuninger M. Dubreuil V. Langenfeld K. Corbeil D. (2005). Release of extracellular membrane particles carrying the stem cell marker prominin-1 (CD133) from neural progenitors and other epithelial cells. J. Cell Sci. 118 (13), 2849–2858. 10.1242/jcs.02439 15976444

[B101] Matta S. M. Hill-Yardin E. L. Crack P. J. (2019). The influence of neuroinflammation in autism spectrum disorder. Brain, Behav. Immun. 79 (April), 75–90. 10.1016/j.bbi.2019.04.037 31029798

[B102] Maxwell M. Bjorkman J. Nguyen T. Sharp P. Finnie J. Paterson C. (2003). Pex13 inactivation in the mouse disrupts peroxisome biogenesis and leads to a zellweger syndrome phenotype. Mol. Cell. Biol. 23 (16), 5947–5957. 10.1128/MCB.23.16.5947-5957.2003 12897163 PMC166343

[B103] Mazzulli J. R. Xu Y. H. Sun Y. Knight A. L. McLean P. J. Caldwell G. A. (2011). Gaucher disease glucocerebrosidase and α-synuclein form a bidirectional pathogenic loop in synucleinopathies. Cell 146 (1), 37–52. 10.1016/j.cell.2011.06.001 21700325 PMC3132082

[B104] McAllister A. K. (2007). Dynamic aspects of CNS synapse formation. Annu. Rev. Neurosci. 30, 425–450. 10.1146/annurev.neuro.29.051605.112830 17417940 PMC3251656

[B105] McCaughey J. Stephens D. J. (2018). COPII-dependent ER export in animal cells: adaptation and control for diverse cargo. Histochem. Cell Biol. 150 (2), 119–131. 10.1007/s00418-018-1689-2 29916038 PMC6096569

[B106] Mengel E. Klünemann H.-H. Lourenço C. M. Hendriksz C. J. Sedel F. Walterfang M. (2013). Niemann-Pick disease type C symptomatology: an expert-based clinical description. Orphanet J. Rare Dis. 8, 166. 10.1186/1750-1172-8-166 24135395 PMC3853996

[B107] Milenkovic I. Jarc J. Dassler E. Aronica E. Iyer A. Adle-Biassette H. (2018). The physiological phosphorylation of tau is critically changed in fetal brains of individuals with Down syndrome. Neuropathol. Appl. Neurobiol. 44 (3), 314–327. 10.1111/nan.12406 28455903

[B108] Mohan A. G. Calenic B. Ghiurau N. A. Duncea-Borca R. M. Constantinescu A. E. Constantinescu I. (2023). The Golgi apparatus: a voyage through time, structure, function and implication in neurodegenerative disorders. Cells 12 (15), 1972–2022. 10.3390/cells12151972 37566051 PMC10417163

[B109] Moore E. M. Migliorini R. Infante M. A. Riley E. P. (2014). Fetal alcohol spectrum disorders: recent neuroimaging findings. Curr. Dev. Disord. Rep. 1 (3), 161–172. 10.1007/s40474-014-0020-8 25346882 PMC4207054

[B110] Mourelatos Z. Adler H. Hirano A. Donnenfeld H. Gonatas J. O. Gonatas N. K. (1990). Fragmentation of the Golgi apparatus of motor neurons in amyotrophic lateral sclerosis revealed by organelle-specific antibodies. Proc. Natl. Acad. Sci. U. S. A. 87 (11), 4393–4395. 10.1073/pnas.87.11.4393 2349244 PMC54116

[B111] Nair A. Greeny A. Rajendran R. Abdelgawad M. A. Ghoneim M. M. Raghavan R. P. (2023). KIF1A-associated neurological disorder: an overview of a rare mutational disease. Pharmaceuticals 16 (2), 147. 10.3390/ph16020147 37259299 PMC9962247

[B112] Nixon R. A. (2013). The role of autophagy in neurodegenerative disease. Nat. Med. 19 (8), 983–997. 10.1038/nm.3232 23921753

[B113] Nuebel E. Morgan J. T. Fogarty S. Winter J. M. Lettlova S. Berg J. A. (2021). The biochemical basis of mitochondrial dysfunction in Zellweger Spectrum Disorder. EMBO Rep. 22 (10), 519911–e52024. 10.15252/embr.202051991 PMC8490991 34351705

[B114] Oliet S. H. R. Mothet J. P. (2009). Regulation of N-methyl-d-aspartate receptors by astrocytic d-serine. Neuroscience 158 (1), 275–283. 10.1016/j.neuroscience.2008.01.071 18358625

[B115] Parenti G. Andria G. Ballabio A. (2015). Lysosomal storage diseases: from pathophysiology to therapy. Annu. Rev. Med. 66, 471–486. 10.1146/annurev-med-122313-085916 25587658

[B116] Park S. Y. Guo X. (2014). Adaptor protein complexes and intracellular transport. Biosci. Rep. 34 (4), e00123–e00390. 10.1042/BSR20140069 24975939 PMC4114066

[B117] Pascual M. Ibáñez F. Guerri C. (2020). Exosomes as mediators of neuron-glia communication in neuroinflammation. Neural Regen. Res. 15 (5), 796–801. 10.4103/1673-5374.268893 31719239 PMC6990780

[B118] Perets N. Hertz S. London M. Offen D. (2018). Intranasal administration of exosomes derived from mesenchymal stem cells ameliorates autistic-like behaviors of BTBR mice. Mol. Autism 9 (1), 57–12. 10.1186/s13229-018-0240-6 30479733 PMC6249852

[B119] Phuyal S. Farhan H. (2021). Want to leave the er? We offer vesicles, tubules, and tunnels. J. Cell Biol. 220 (6), e202104062–e202104064. 10.1083/jcb.202104062 33999114 PMC8129806

[B120] Platt F. M. d'Azzo A. Davidson B. L. Neufeld E. F. Tifft C. J. (2018). Lysosomal storage diseases. Nat. Rev. Dis. Prim. 4 (1), 27. 10.1038/s41572-018-0025-4 30275469

[B121] Pluta R. Ułamek-Kozioł M. Januszewski S. Czuczwar S. J. (2018). Exosomes as possible spread factor and potential biomarkers in Alzheimer's disease: current concepts. Biomarkers Med. 12 (9), 1025–1033. 10.2217/bmm-2018-0034

[B122] Polenghi M. Taverna E. (2023). Intracellular traffic and polarity in brain development. Front. Neurosci. 17, 1172016. 10.3389/fnins.2023.1172016 37859764 PMC10583573

[B123] Powers J. M. Moser H. W. (1998). Peroxisomal disorders: genotype, phenotype, major neuropathologic lesions, and pathogenesis. Brain Pathol. 8 (1), 101–120. 10.1111/j.1750-3639.1998.tb00139.x 9458170 PMC8098283

[B124] Pressey S. N. R. Smith D. A. Wong A. M. S. Platt F. M. Cooper J. D. (2012). Early glial activation, synaptic changes and axonal pathology in the thalamocortical system of Niemann-Pick type C1 mice. Neurobiol. Dis. 45 (3), 1086–1100. 10.1016/j.nbd.2011.12.027 22198570 PMC3657200

[B125] Pujol A. Ferrer I. Camps C. Metzger E. Hindelang C. Callizot N. (2004). Functional overlap between ABCD1 (ALD) and ABCD2 (ALDR) transporters: a therapeutic target for X-adrenoleukodystrophy. Hum. Mol. Genet. 13 (23), 2997–3006. 10.1093/hmg/ddh323 15489218

[B126] Rabouille C. (2017). Pathways of unconventional protein secretion. Trends Cell Biol. 27 (3), 230–240. 10.1016/j.tcb.2016.11.007 27989656

[B127] Rajendran L. Honsho M. Zahn T. R. Keller P. Geiger K. D. Verkade P. (2006). Alzheimer's disease β-amyloid peptides are released in association with exosomes. Proc. Natl. Acad. Sci. U. S. A. 103 (30), 11172–11177. 10.1073/pnas.0603838103 16837572 PMC1544060

[B128] Raposo G. Stoorvogel W. (2013). Extracellular vesicles: exosomes, microvesicles, and friends. J. Cell Biol. 200 (4), 373–383. 10.1083/jcb.201211138 23420871 PMC3575529

[B129] Rava A. La Rosa P. Palladino G. Dragotto J. Totaro A. Tiberi J. (2022). The appearance of phagocytic microglia in the postnatal brain of Niemann Pick type C mice is developmentally regulated and underscores shortfalls in fine odor discrimination. J. Cell. Physiol. 237 (12), 4563–4579. 10.1002/jcp.30909 36322609 PMC7613956

[B130] Rocha E. M. Smith G. A. Park E. Cao H. Graham A. R. Brown E. (2015). Sustained systemic glucocerebrosidase inhibition induces brain α-synuclein aggregation, microglia and complement C1q activation in mice. Antioxidants Redox Signal. 23 (6), 550–564. 10.1089/ars.2015.6307 PMC4544823 26094487

[B131] Saeedi S. Israel S. Nagy C. Turecki G. (2019). The emerging role of exosomes in mental disorders. Transl. Psychiatry 9 (1), 122. 10.1038/s41398-019-0459-9 30923321 PMC6438960

[B132] Saftig P. Klumperman J. (2009). Lysosome biogenesis and lysosomal membrane proteins: trafficking meets function. Nat. Rev. Mol. Cell Biol. 10 (9), 623–635. 10.1038/nrm2745 19672277

[B133] Saman S. Kim W. H. Raya M. Visnick Y. Miro S. Saman S. (2012). Exosome-associated tau is secreted in tauopathy models and is selectively phosphorylated in cerebrospinal fluid in early Alzheimer disease. J. Biol. Chem. 287 (6), 3842–3849. 10.1074/jbc.M111.277061 22057275 PMC3281682

[B134] Santos M. J. Quintanilla R. A. Toro A. Grandy R. Dinamarca M. C. Godoy J. A. (2005). Peroxisomal proliferation protects from β-amyloid neurodegeneration. J. Biol. Chem. 280 (49), 41057–41068. 10.1074/jbc.M505160200 16204253

[B135] Sapir T. Frotscher M. Levy T. Mandelkow E.-M. Reiner O. (2012). Tau's role in the developing brain: implications for intellectual disability. Hum. Mol. Genet. 21 (8), 1681–1692. 10.1093/hmg/ddr603 22194194

[B136] Sasabe J. Suzuki M. Imanishi N. Aiso S. (2014). Activity of D-amino acid oxidase is widespread in the human central nervous system. Front. Synaptic Neurosci. 6 (JUN), 14–10. 10.3389/fnsyn.2014.00014 24959138 PMC4050652

[B137] Schaefer M. K. E. Schmalbruch H. Buhler E. Lopez C. Martin N. Guénet J. L. (2007). Progressive motor neuronopathy: a critical role of the tubulin chaperone TBCE in axonal tubulin routing from the Golgi apparatus. J. Neurosci. 27 (33), 8779–8789. 10.1523/JNEUROSCI.1599-07.2007 17699660 PMC6672183

[B138] Scott C. Ioannou Y. A. (2004). The NPC1 protein: structure implies function. Biochimica Biophysica Acta - Mol. Cell Biol. Lipids 1685 (1–3), 8–13. 10.1016/j.bbalip.2004.08.006 15465421

[B139] Segev N. Tokarev A. A. Alfonso A. Segev N. (2009). Overview of intracellular compartments and trafficking pathways. in Trafficking inside cells: pathways, mechanisms and regulation, 3–14.

[B140] Senanayake V. Goodenowe D. B. (2019). Plasmalogen deficiency and neuropathology in Alzheimer's disease: causation or coincidence? Alzheimer's Dementia Transl. Res. Clin. Interventions 5, 524–532. 10.1016/j.trci.2019.08.003 PMC6804645 31650009

[B141] Sharma P. Mesci P. Carromeu C. McClatchy D. R. Schiapparelli L. Yates J. R. (2019). Exosomes regulate neurogenesis and circuit assembly. Proc. Natl. Acad. Sci. U. S. A. 116 (32), 16086–16094. 10.1073/pnas.1902513116 31320591 PMC6689941

[B142] Shima D. T. Scales S. J. Kreis T. E. Pepperkok R. (1999). Segregation of COPI-rich and anterograde-cargo-rich domains in endoplasmic-reticulum-to-Golgi transport complexes. Curr. Biol. 9 (15), 821–824. 10.1016/S0960-9822(99)80365-0 10469566

[B143] Singh I. (1997). Biochemistry of peroxisomes in health and disease. Mol. Cell. Biochem. 167 (1–2), 1–29. 10.1023/a:1006883229684 9059978

[B144] Soo K. Y. Halloran M. Sundaramoorthy V. Parakh S. Toth R. P. Southam K. A. (2015). Rab1-dependent ER–Golgi transport dysfunction is a common pathogenic mechanism in SOD1, TDP-43 and FUS-associated ALS. Acta Neuropathol. 130 (5), 679–697. 10.1007/s00401-015-1468-2 26298469

[B145] Spang A. Schekman R. (1998). Reconstitution of retrograde transport from the Golgi to the ER *in vitro* . J. Cell Biol. 143 (3), 589–599. 10.1083/jcb.143.3.589 9813082 PMC2148153

[B146] Steinberg S. J. Dodt G. Raymond G. V. Braverman N. E. Moser A. B. Moser H. W. (2006). Peroxisome biogenesis disorders. Biochimica Biophysica Acta - Mol. Cell Res. 1763 (12), 1733–1748. 10.1016/j.bbamcr.2006.09.010 17055079

[B147] Taverna E. Götz M. Huttner W. B. (2014). The cell biology of neurogenesis: toward an understanding of the development and evolution of the neocortex. Annu. Rev. cell Dev. Biol. 30, 465–502. 10.1146/annurev-cellbio-101011-155801 25000993

[B148] Taverna E. Mora-Bermúdez F. Strzyz P. J. Florio M. Icha J. Haffner C. (2016). Non-canonical features of the Golgi apparatus in bipolar epithelial neural stem cells. Sci. Rep. 6 (January), 1–22. 10.1038/srep21206 26879757 PMC4754753

[B149] Todd A. G. Lin H. Ebert A. D. Liu Y. Androphy E. J. (2013). COPI transport complexes bind to specific RNAs in neuronal cells. Hum. Mol. Genet. 22 (4), 729–736. 10.1093/hmg/dds480 23175440 PMC3988478

[B150] Tracy T. E. Madero-Pérez J. Swaney D. L. Chang T. S. Moritz M. Konrad C. (2022). Tau interactome maps synaptic and mitochondrial processes associated with neurodegeneration. Cell 185 (4), 712–728.e14. 10.1016/j.cell.2021.12.041 35063084 PMC8857049

[B151] Tsilioni I. Theoharides T. C. (2018). Extracellular vesicles are increased in the serum of children with autism spectrum disorder, contain mitochondrial DNA, and stimulate human microglia to secrete IL-1β. J. Neuroinflammation 15 (1), 239–248. 10.1186/s12974-018-1275-5 30149804 PMC6112123

[B152] Uzman A. (2003). Molecular biology of the cell: Alberts, B., Johnson, A., Lewis, J., Raff, M., Roberts, K., and Walter, P. USA: John Wiley & Sons Inc.

[B153] Volpe J. J. Adams R. D. (1972). Cerebro-hepato-renal syndrome of Zellweger: an inherited disorder of neuronal migration. Acta Neuropathol. 20 (3), 175–198. 10.1007/BF00686900 5043999

[B154] Washbourne P. (2015). Synapse assembly and neurodevelopmental disorders. Neuropsychopharmacology 40 (1), 4–15. 10.1038/npp.2014.163 24990427 PMC4262893

[B155] Wei Y. Wang D. Jin F. Bian Z. Li L. Liang H. (2017). Pyruvate kinase type M2 promotes tumour cell exosome release via phosphorylating synaptosome-associated protein 23. Nat. Commun. 8, 14041. 10.1038/ncomms14041 28067230 PMC5228053

[B156] Wood P. L. Locke V. A. Herling P. Passaro A. Vigna G. B. Volpato S. (2016). Targeted lipidomics distinguishes patient subgroups in mild cognitive impairment (MCI) and late onset alzheimer's disease (LOAD). BBA Clin. 5, 25–28. 10.1016/j.bbacli.2015.11.004 27051586 PMC4802395

[B157] Wu S. Villegas N. C. H. Sirkis D. W. Thomas-Wright I. Wade-Martins R. Schekman R. (2023). Unconventional secretion of α-synuclein mediated by palmitoylated DNAJC5 oligomers. ELife 12, 1–29. 10.7554/eLife.85837 PMC9876576 36626307

[B158] Xu X. Kedlaya R. Higuchi H. Ikeda S. Justice M. J. Setaluri V. (2010). Mutation in archain 1, a subunit of COPI coatomer complex, causes diluted coat color and Purkinje cell degeneration. PLoS Genet. 6 (5), e1000956. 10.1371/journal.pgen.1000956 20502676 PMC2873907

[B159] Yang X. Y. Zhou X. Y. Wang Q. Q. Li H. Chen Y. Lei Y. P. (2013). Mutations in the COPII vesicle component gene SEC24B are associated with human neural tube defects. Hum. Mutat. 34 (8), 1094–1101. 10.1002/humu.22338 23592378

[B160] Yu X. Breitman M. Goldberg J. (2012). A Structure-based mechanism for Arf1-dependent recruitment of coatomer to membranes. Cell 148 (3), 530–542. 10.1016/j.cell.2012.01.015 22304919 PMC3285272

[B161] Zanetti G. Prinz S. Daum S. Meister A. Schekman R. Bacia K. (2013). The structure of the COPII transport-vesicle coat assembled on membranes. ELife 2013 (2), 009511–e1015. 10.7554/eLife.00951 PMC3778437 24062940

[B162] Zeuschner D. Geerts W. J. C. van Donselaar E. Humbel B. M. Slot J. W. Koster A. J. (2006). Immuno-electron tomography of ER exit sites reveals the existence of free COPII-coated transport carriers. Nat. Cell Biol. 8 (4), 377–383. 10.1038/ncb1371 16531996

[B163] Zhang M. Schekman R. (2013). Cell biology. Unconventional secretion, unconventional solutions. Science 340 (6132), 559–561. 10.1126/science.1234740 23641104

[B164] Zhang X. Szabo E. Michalak M. Opas M. (2007). Endoplasmic reticulum stress during the embryonic development of the central nervous system in the mouse. Int. J. Dev. Neurosci. 25 (7), 455–463. 10.1016/j.ijdevneu.2007.08.007 17913437

[B165] Zhou J. Benito-Martin A. Mighty J. Chang L. Ghoroghi S. Wu H. (2018). Retinal progenitor cells release extracellular vesicles containing developmental transcription factors, microRNA and membrane proteins. Sci. Rep. 8 (1), 2823–2915. 10.1038/s41598-018-20421-1 29434302 PMC5809580

[B166] Zhou J. Flores-Bellver M. Pan J. Benito-Martin A. Shi C. Onwumere O. (2021). Human retinal organoids release extracellular vesicles that regulate gene expression in target human retinal progenitor cells. Sci. Rep. 11 (1), 21128–21217. 10.1038/s41598-021-00542-w 34702879 PMC8548301

[B167] Zou W. Lv Y. Zhang S. Li L. Sun L. Jiao J. (2024). Lysosomal dynamics regulate mammalian cortical neurogenesis. Dev. Cell 59 (1), 64–78.e5. 10.1016/j.devcel.2023.11.021 38103552

